# Influence of chromium hyperdoping on the electronic structure of CH_3_NH_3_PbI_3_ perovskite: a first-principles insight

**DOI:** 10.1038/s41598-018-20851-x

**Published:** 2018-02-06

**Authors:** Gregorio García, Pablo Palacios, Eduardo Menéndez-Proupin, Ana L. Montero-Alejo, José C. Conesa, Perla Wahnón

**Affiliations:** 1 Instituto de Energía Solar, ETSI Telecomunicación, Universidad Politécnica de Madrid, Ciudad Universitaria, s/n, 28040 Madrid, Spain; 2 Departamento de Tecnología Fotónica y Bioingeniería, ETSI Telecomunicación, Universidad Politécnica de Madrid, Ciudad Universitaria, s/n, 28040 Madrid, Spain; 3 Departamento de Física aplicada a las Ingenierías Aeronáutica y Naval, ETSI Aeronáutica y del Espacio, Universidad Politécnica de Madrid, Pz. Cardenal Cisneros, 3, 28040 Madrid, Spain; 4 Grupo de Modelización de Materiales, Departamento de Física, Facultad de Ciencias, Universidad de Chile, Las Palmeras, 3425, 780-0003 Ñuñoa Santiago, Chile; 5 Instituto de Catálisis y Petroleoquímica, Consejo Superior de Investigaciones Científicas, Marie Curie 2, 28049 Madrid, Spain

## Abstract

Organic-inorganic hybrid halide perovskites compounds are emerging as new materials with great potential for efficient solar cells. This paper explores the possibility of increasing their photovoltaic efficiency through sub-bandgap absorption by way of the in gap band (IGB) concept. Thus, we assess the formation of an in gap band as well as its effect on the absorption features of Organic-inorganic hybrid halide perovskites CH_3_NH_3_PbI_3_ (MAPI). For this task, we use density functional theory (DFT) as well as many-body perturbation methods along to spin-orbit coupling (SOC) to study structural, energetic and electronic properties of partially Cr-substituted MAPI perovskites (CH_3_NH_3_Pb_1−x_Cr_x_I_3_). Our results reveal that Cr replacement does not lead to an important cell distortion, while the energetic of the substitution process evidences the possibility of obtaining Cr-substituted perovskite. The analysis of the electronic structure shows that Cr *3d-*orbitals induce new electronic states in the host semiconductor bandgap, which fulfill the requirements to be considered as an IGB. Precise many-body perturbation methods in *G*_0_*W*_0_ approach provided an accurate description on the electronic structures as well as the position of the IGB. In short, Pb replacement by Cr could be useful for improved absorption features through new sub-bandgap transitions across the in gap band.

## Introduction

**Figure 1:**
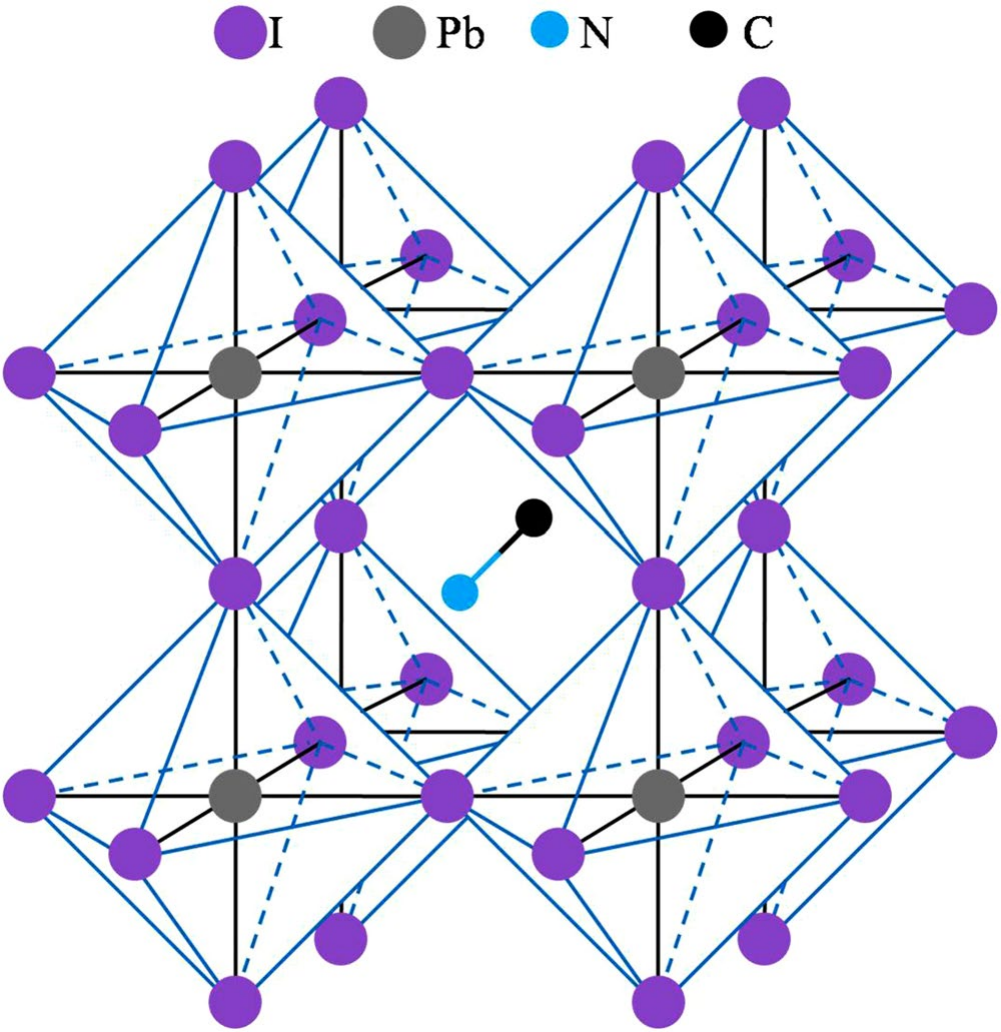
Crystal structure of MAPI perovskite. H atoms were omitted for clarity.

Currently, there is a considerable interest in the use of organic-inorganic hybrid perovskites (with general formula AMX_3_, A = organic cation, M = metal, X = halide) to develop low cost and high efficiency photovoltaic devices due to their adequate optical and electronic properties^1,2^. Of these, methylammonium lead trihalide perovskite with A = CH_3_NH_3_, M = Pb and X = I (also known as MAPI, see Fig. 1) has attracted significant attention because it has allowed reaching power conversion efficiencies (PCEs) ≈20% or even higher in the few years passed since its application was first reported in 2009^2–5^. As matter of fact, MAPI exhibits adequate properties as a solar absorber, such as: a direct bandgap of ≈1.6 eV allowing a very broad absorption range over the entire visible light region^1,4,6^, small exciton binding energies^6^, high levels of defect self-regulation^7^, long charge carrier diffusion lengths^8,9^, and excellent charge carrier mobilities^10,11^. In common thin-film solar cells materials such as CdTe or GaAs, the conduction band minimum (CBM) is mostly due to cation *s* and anion *s* states, while the valence band maximum (VBM) is mainly formed by anion *p* states. However, the electronic structure of MAPI perovskite is inverted: here the VBM is characterized by hybridized Pb *(6s)* and I *(5p)*, while the CBM is mainly composed of Pb *(6p)* states. In this sense, *ab initio* calculations have pointed out that the direct bandgap between Pb (6*s*)-I (*5p*) valence bands and Pb (*6p*) conduction band (this transition is Pb dominated) is the key parameter for an improved light absorption^12–14^. MAPI also allows to be solution-processed leading to a low cost-fabrication^10^, it performs well with a wide range of hole and electron contact materials^5^, and experiences little to no performance degradation over extended outdoor testing^15^. Then, MAPI is a promising component for solar energy conversion devices in the future^4,5,12,16,17^.

Although perovskite solar cells have developed faster progress, the efficiency of perovskite-based (mainly MAPI-based) solid-state photovoltaics is still much lower than the single gap Schokley-Queisser limit. The theoretical maximum efficiency limit has recently predicted in the range of 25–27% based on Shockley-Queissers model^18^, while conversion efficiencies ≈33% can be obtained for the current state of single junction solar cells with a bandgap energy (*E*_*g*_) of 1.34 eV^19^. To further explore the potentials and increase the efficiency of perovskites solar cells, a great amount of work has been carried out in the last in the last years. Most of these works are focused on the ability of fine-tune the electronic properties of AMX_3_ perovskites through the chemical composition. For instance, the bandgap can be shifted to the visible or to the near-infrared spectrum by using mixed-halide perovskites with different ratio of halides^20–22^. Respect to the role of the organic cation, the replacement of conventional methylammonium cation with formamidinium^23^, cesium^24^ or their mixtures^25–28^ led to an improved stability. Concerning the metal, the main efforts are devoted to the replacement of Pb atom since lead compounds have high toxicity and environmental problems^1^. For example, N. K. Noel *et al*. synthesized a lead free perovskite where Pb was replaced with Sn, but with a lower PCE (≈6%)^29^. K. Wang *et al*. studied the role of the metal M (M = Pb, Sn, Ge, and Sr) by first principles calculations^16^. However, it is largely unknown how partial replacement of Pb atoms affect material properties and photovoltaic performance. For instance, the partial replacement of Pb by Sn results in a gap narrowing with efficiencies up to 15.1%^30,31^. M. T. Klug *et al*. assessed the effect of a partial replacement of Pb with alternative divalent metal species (M = Co, Cu, Fe, Mg, Mn, Ni, Sn, Sr and Zn) on the photovoltaic performance and optical properties of MAPI. Their results suggest that the perovskite material is tolerant to partial replacement of Pb by most of the species considered, with lead-cobalt mixture (with less than 6% of Pb atom replaced) leading to the highest PCE (above 16%)^17^.

Besides this, there are also a wide range of approaches applied to common semiconductors that can potentially improve the photovoltaic efficiency^32^. One possible way of increasing PCE is through sub-bandgap absorption by way of the in gap band (IGB) concept. In those materials with an IGB, a partially filled narrow band (IGB, also known as intermediate band) is located between the valence and conduction bands (VB and CB, respectively) of the host semiconductors. Thus, IGB materials could improve the PCE through the absorption of two extra sub-bandgap photons (from the VB to the IGB and from there to the CB). This would result in the creation of additional electron-hole pairs, and in principle, in an increase in photocurrent without a decrease in open-circuit voltage. A cell based on such approach could reach theoretical efficiencies up to 63.1%^33^. In addition, an IGB should fulfill some requirements to really improve photovoltaic efficiency^33,34^ (*i*) small dispersion, although without becoming a discrete level; (*ii*) be narrow enough to be well isolated from the valence and conduction bands, to avoid the thermalization between the IGB and the VB or CB; (*iii*) should be partially filled, which allows comparable rates for the two possible absorption processes across the IGB; (*iv*) high concentrations of IGB states to both produce high absorption coefficient in the new absorptions and avoid non radiative recombination obtained through the formation of a highly delocalized energy band. Among different strategies to carry out the IGB, in our group we have extensively studied the formation of an IGB through the replacement of some cations by a transition metal. In this way, *d*-orbitals of the transition metal might be located within the bandgap of semiconductors allowing the formation of the isolated energy band, while the filling of the IGB would be modulated through the electronic configuration of the transition metal. The formation of this band is controlled by the spatial symmetry of the *d*-orbitals of the transition metal, which is different from those *s* and *p*-type orbitals that form the VBs and the CBs in commonly used semiconductors. This approach has been widely studied to improve the efficiency of common semiconductors with photovoltaic performance such as GaP^35–37^, CuGaS_2_^38–43^, thiospinels^44,45^, sulfides like SnS_2_^46^ or Si^47,48^. The formation of an IGB in CdTe through the insertion of other elements such as Bi or Sn has been also reported^49,50^.

To our knowledge, proposals for the creation of an IGB in MAPI-type perovskites are rather scarce. Bearing in mind that around the fundamental gap of MAPI, only I and Pb contribute to the density of states^51^, our work explores the effect of partial replacing of Pb with a transition metal. This type of substitution has been attempted recently^52–54^, but only M. D. Sampson *et al*.^53^ mention the interest of an IGB, and in any case none of these works seems to result in a partially filled IGB. Hence, in order to rationalize the formation of the in gap band and its effect on electronic properties of MAPI perovskite, a detailed *ab initio* computational study of structural, energetic and electronic properties of different Cr-substituted MAPI perovskites (CH_3_NH_3_Pb_1−x_Cr_x_I_3_) as well as Cr concentration (x = 0.25, 0.125) is here presented. Cr-dopant element has been selected since this transition metal fulfills the requirements to form a partially occupied in gap band. After the replacement of Pb by Cr, the transition metal will be located at a CrI_6_ octahedral environment. This octahedral symmetry would cause a split of the Cr 3*d*-orbitals into a low-energy *t*_*2g*_ triplet (due to *d*_*xy*_, *d*_*xz*_ and *d*_*yz*_) and a high-energy *e*_*g*_ doublet (${d}_{{z}^{2}}$ and ${d}_{{x}^{2}-{y}^{2}}$). In a high spin-configuration (which is common for transitions metals prior to Mn), *e*_*g*_ states will be partially filled, since Cr(II) is a *3d*^4^ ion. In addition, *e*_*g*_ states own larger spatial extension, which facilitates the orbital delocalization as well as the formation of a delocalized band, which may be thus partially occupied.It is well known that *ab initio* computational approaches are capable to provide a deep knowledge of the electronic structure of perovskites, which is fundamentally required for further progress in photovoltaic efficiencies^13,16,55–57^. However, while common density functional methods (DFT) based on the GGA (Generalized Gradient approximation) scheme for the exchange-correlation functional are accurate for the study of structural properties and relative stabilities, they fail to correctly predict electronic structure and related properties, such as the bandgap, of semiconductors^58^. This behavior has been also reported for perovskites^13,16,55–57^. Hence, here we employ many-body perturbation theory in *GW* approximation to calculate quasiparticle self-energy corrections for the electronic states, which provides accurate results for a wide range of materials^59^. Recently, *GW* approach along to explicit spin-orbit coupling (SOC) method (for the adequate treatment of Pb atom) has been applied to MAPI perovskites, yielding bandgap values that are in very good agreement with experiments^13,55,60^. Thus, the main goal of this work is the study, through DFT methods and many body techniques like the *GW* approach, of the structure, stability and electronic properties of the above mentioned Cr-substituted MAPI perovskite. We find that partial replacement of Pb with Cr in MAPI perovskite leads to the formation of an IGB with the desired properties, which point out that the photovoltaic efficiency could be improved due to the absorption of two extra photons thanks to the in gap band.

## Results and Discussion

The study of MAPI and Cr@MAPI perovskites is divided into three sections aimed at assessing the main effects of Pb replacement by Cr on the structural and electronic features of the native MAPI from an atomistic point of view. The first section analyzes the main structural properties of MAPI and Cr@MAPI perovskites, as well as the changes due to the presence of Cr. The second section provides an analysis of the formation energies and stability of Cr-substituted perovskite. The last section reports an insight into the electronic structure. Thus, the effects due to Cr atom on the electronic structure of MAPI perovskite and their relationship to the improvement of sunlight absorption are discussed. In this section, we not only investigate the electronic structure of MAPI and Cr@MAPI perovskites trying to deepen on the influence of Cr atom, but also we highlight the need to go beyond standard DFT methods to accurately predict electronic structure related properties.

### Main structural parameters

**Figure 2:**
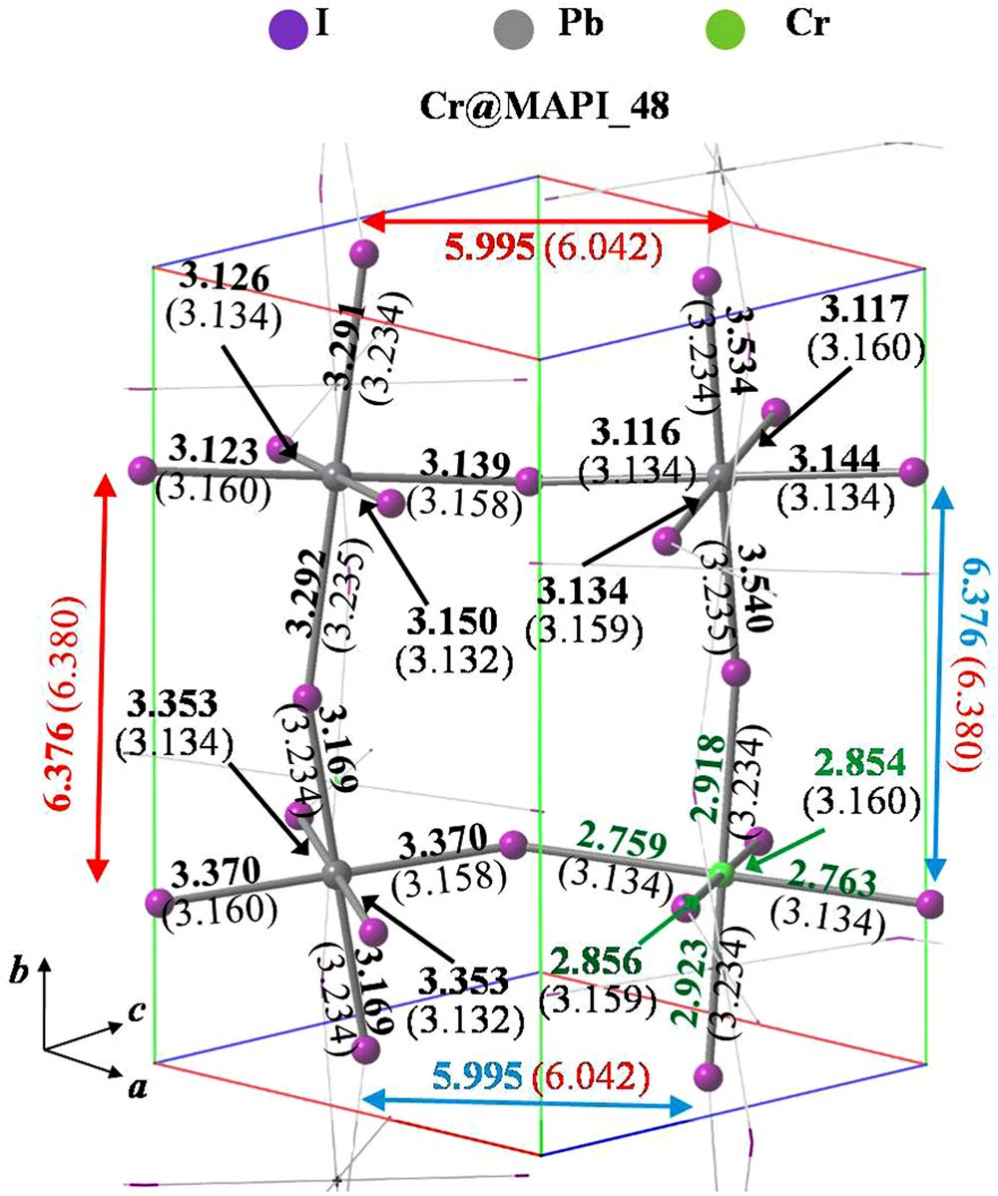
Optimized structures of Cr@MAPI_48 perovskite, along with bond lengths of I-Pb (black), I-Cr (green), Pb-Pb (red) and Pb-Cr (blue). Values in parenthesis stand for those bond lengths for the optimized structures of the native MAPI. For clarity, Pb-Pb and Pb-Cr bond distances are displayed outside of the unit cell. Methylammonioum cations were omitted for simplicity.

In the crystal structure of MAPI perovskite, each methylammonium cation is surrounded by eight PbI_6_ octahedra sharing corners (see Fig. 1). Table 1 collects lattice cell parameters and some representative bond lengths. As seen, PBEsol functional leads to lattice parameters and bond lengths of MAPI in good agreement with the experimental data found for the low temperature orthorhombic phase (the departure of the optimized unit cell from experimental data lies in the range 0.3–2.0% and 0.2–6.4% for lattice parameters and bond lengths, respectively). The largest differences (4.5–6.4%) are found for C-N bond distance, which is slightly reduced respect to experimental bond length. Nonetheless, C-N theoretical bond length lies in the range of a typical C-N bond. The average bond length of I-Pb in PbI_6_ octahedra is found to be ≈3.19 Ǻ. Our results also reproduce the bond length alternation of I-Pb bonds (see Figs 2 and S1 for more details)^61^. In this sense, I-Pb bonds can be grouped into three pairs with bond lengths ≈3.36 Ǻ, 3.23 Ǻ and 3.16 Ǻ. In our previous works^51,62^, the structure of MAPI perovskite was optimized by using three density functional including dispersion interactions, *i.e*., vdW-DF2, optB88-vdW and optB86b-vdW, as well as the PBE functional. As results, van der Waals functionals accurately reproduce the unit cell volume^51^. Although PBEsol functional provides results slightly worse in comparison to van der Waals functionals, our simulations confirm its capability to study structural properties of hybrid lead halide perovskites along to a reduced computational cost.

**Table 1:** Lattice parameters, bond distances and cell volumes obtained from optimized crystal structure of MAPI perovskite (CH_3_NH_3_PbI_3_) for the unit cell with 48 atoms (MAPI_48), and two larger 96-atoms supercells: (a) with *a ≈ b ≈ c* (MAPI_96*a*) and (b) 2 × 1 × 1 supercell, *i.e*. replicating lattice parameter *a* (MAPI_96*b*). Values in parentheses stand for Relative errors between experimental and theoretical results.

	MAPI (CH_3_NH_3_PbI_3_)			
	MAPI_48^a^	MAPI_48	MAPI_96*a*	MAPI_96*b*
Volume/Ǻ^3^	951.01	931.53 (2.04%)	1907.65 (0.30%)	1913.62
*a*/Ǻ	8.836	8.66 (1.99%)	12.16	17.72 (0.27%)
*b*/Ǻ	12.580	12.76 (1.43%)	12.911	12.69 (0.87%
*c*/Ǻ	8.555	8.43 (1.46)	12.15	8.51 (0.95)
d(C-N)^b^/Ǻ	1.571	1.50 (4.52%)	1.47 (6.4)	1.48 (5.79%)
d(I-Pb)^b^/Ǻ	3.176	3.17 (0.19%)	3.19 (0.44)	3.20 (0.76)
d(H-I)^b^/Ǻ		2.62	2.58	2.58
d(Pb-Pb)^b^/Ǻ		6.21	6.17	6.22

^a^Experimental data taken from Baikie *et al*.^88^.

^b^Average distances over each type of bonds are reported. For more details, see Figs 2 and S1.

Figure 2 and Table 2 report the optimized structure of Cr@MAPI perovskites (CH_3_NH_3_Pb_1−x_Cr_x_I_3_) along to the main structural parameters for x = 0.25 (Cr@MAPI_48) and x = 0.125 (Cr@MAPI_96*a* and Cr@MAPI_96*b*). Broadly, the lattice parameters of Cr@MAPI do not suffer important changes upon Cr presence. For Cr@MAPI_48, the final structure has a volume that is smaller by an amount of 14.73 Ǻ^3^ (*i.e*. 0.31 Ǻ^3^ per atom). Lattice parameters *b* and *c* remain practically unaltered, while lattice parameter *a* decreases to 8.55 Ǻ (*i.e*. 0.11 Ǻ shorter). Both Pb and Cr atoms are surrounded by an octahedron made up of six I atoms, with average distances equal to 3.21 Ǻ and 2.85Ǻ for I-Pb and I-Cr bonds, respectively. For I-Cr bonds, in the CrI_6_ octahedron, there is also a bond length alternation, wherein I-Cr bonds can be grouped into three pairs with bond lengths ≈2.92 Ǻ, 2.86 Ǻ and 2.76 Ǻ, which entails a decrease of ≈0.40 Ǻ respect to I-Pb bonds in the native MAPI perovskite (see Fig. 2). Optimized I-Cr bond lengths here reported are similar to those previously found for CsCrI_3_, with an average I-Cr bond length equal to 2.89 Ǻ in its low temperature form^63^. In this compound, each Cr atom is also surrounded by an octahedron of I atoms. Alike, I-Cr bond in CsCrI_3_ are also grouped into three pairs with bond lengths of 3.05 Ǻ, 2.85 Ǻ and 2.76 Ǻ. As concerns the I-Pb bonds, their average bond distance is slightly lengthened (0.04 Ǻ). As seen in Fig. 2, the Pb-I distances involved in Pb-I-Cr bonds are the most affected ones, being incremented by an amount ≈0.26 Ǻ. Finally, Pb-Pb and Pb-Cr distances can be used as a measurement of the void wherein methylammonium cation is placed. Both Pb-Pb and Pb-Cr provide a distance of 6.19 Ǻ, slightly lower than Pb-Pb distance found in MAPI_48 (6.21 Ǻ). For the sake of the brevity a detailed analysis of I-Pb and I-Cr bonds is here avoided for Cr@MAPI_96*a* and Cr@MAPI_96*b*, because similar trends are obtained irrespective of the Cr concentration (see Table 2 and Fig. S1). The main differences are noted for Pb-Pb and Pb-Cr distances. The Pb-Pb distance is increased up to 6.23 Ǻ and 6.28 Ǻ for Cr@MAPI_96*a* and Cr@MAPI_96*b*, respectively (6.17 Ǻ and 6.22 Ǻ for MAPI_96*a* and MAPI_96*b*, respectively). Meanwhile, the Pb-Cr distance is something smaller than Pb-Cr distance in Cr@MAPI_48. Based on here reported results, it can be concluded that the presence of Cr atom does not lead to an important cell distortion, which decreases at long range and upon smaller Cr quantities.

**Table 2:** Lattice parameters, bond distances and cell volumes obtained from optimized crystal structure of Cr-substituted MAPI perovskite (CH_3_NH_3_Pb_1−x_Cr_x_I_3_) for the unit cell with 48 atoms (Cr@MAPI_48, x = 0.25), and two larger 96-atoms supercells: (a) with *a ≈ b ≈ c* (Cr@MAPI_96*a*, x = 0.125) and (b) 2 × 1 × 1 supercell, *i.e*. replicating lattice parameter *a* (Cr@MAPI_96*b*, x = 0.125).

	Cr@MAPI (CH_3_NH_3_Pb_1−x_Cr_x_I_3_)		
	Cr@MAPI_48	Cr@MAPI_96*a*	Cr@MAPI_96*b*
	x = 0.25	x = 0.125	x = 0.125
Volume/Ǻ^3^	916.80	1896.59	1913.10
*a*/Ǻ	8.55	12.13	17.82
*b*/Ǻ	12.75	12.89	12.72
*c*/Ǻ	8.41	12.13	8.44
d(C-N)^a^/Ǻ	1.48	1.47	1.48
d(I-Pb) ^a^/Ǻ	3.21	3.23	3.24
d(I-Cr)^a^/Ǻ	2.85	2.85	2.85
d(H-I)^a^/Ǻ	2.65	2.57	2.57
d(Pb-Pb)^a^/Ǻ	6.19	6.23	6.28
d(Pb-Cr)^a^/Ǻ	6.19	6.17	6.09

^a^Average distances over each type of bonds are reported. For more details, see Figs 2 and S1.

### Energetics

To find out the ease of formation of Cr@MAPI perovskite material here studied, we have considered the energetic cost of incorporating Cr to the native MAPI perovskite by the following substitution reaction:

1 $${{\rm{CH}}}_{3}{{\rm{NH}}}_{3}{{\rm{PbI}}}_{3}{+\text{xCrI}}_{2}\to {{\rm{CH}}}_{3}{{\rm{NH}}}_{3}{{\rm{Pb}}}_{1-{\rm{x}}}{\rm{CrxI3}}+{{\rm{xPbI}}}_{2}$$

To compute the corresponding energies, 48-atom cells have been used for both perovskite structures, and the reported crystal structures for PbI_2_^64^ and CrI_2_^65,66^ have been used. In all cases the structures have been relaxed at the PBEsol level (see above). The energy balance of the mentioned reaction for x = 0.25 is then:

2 $${\rm{\Delta }}E={E}_{[Cr@MAPI\_48]}+{E}_{[PbI2]}-({E}_{[MAPI\_48]}+{E}_{[CrI2]})=+\,0.38\,{\rm{eV}}$$

where *E*_*[Cr@MAPI_48]*_, *E*_*[MAPI_48]*_, *E*_*[PbI2]*_ and *E*_*[CrI2]*_ stand for the total energies of Cr@MAPI_48, MAPI_48, PbI_2_ and CrI_2_, respectively. The positive value means that substituting Pb by Cr is not favoured energetically. However, disorder entropy will be favouring this process, in more or less extend depending on temperature. In any case this value is smaller than those previously reported other in other in gap band materials obtained through the hyperdoping with transition metals. For example, the formation energy here calculated for Cr@MAPI_48 is smaller than that computed for the replacement of Ga by Mn in GaAs (*ΔE* = +2.05 eV)^67^, Ga by Ti in GaP (*ΔE* = +2.05 eV)^67^, Ga by Cr in CuGaS_2_ (*ΔE* = +0.61 eV)^68^ or Ga by Ti in CuGaS_2_ (*ΔE* = +0.2–0.8 eV)^68^. The synthesis of these materials has been experimentally achieved^69–72^, pointing out that small positive formation energies, when translated into free energies including entropy, can be reduced or be made negative through the adequate experimental conditions, or alternatively that the materials can be made if the preparations are controlled by kinetic rather than thermodynamic factors. Therefore, our results suggest that Cr-substituted perovskite should be feasible by using the adequate synthesis methods, even though a more detailed thermodynamic assessment may be undertaken in a future work.

### Electronic Structure

**Figure 3:**
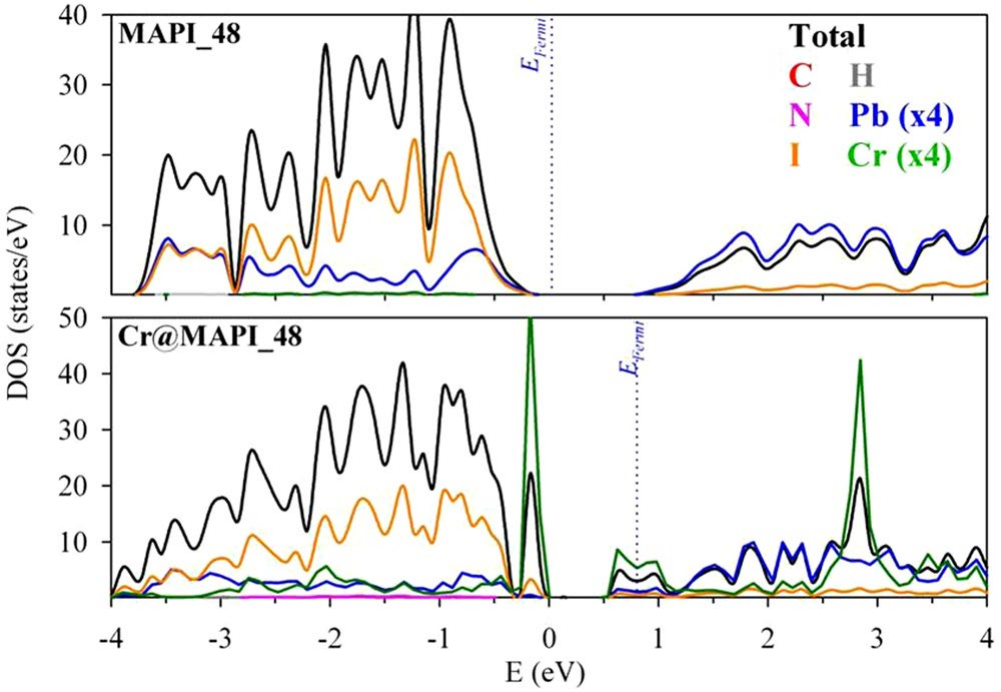
Projected Density of States of MAPI_48 and Cr@MAPI_48 calculated with PBEsol +SOC. The zero of energy has been set at the valence band energy, while blue dotted line is representing the Fermi level (*E*_*Fermi*_).

**Figure 4:**
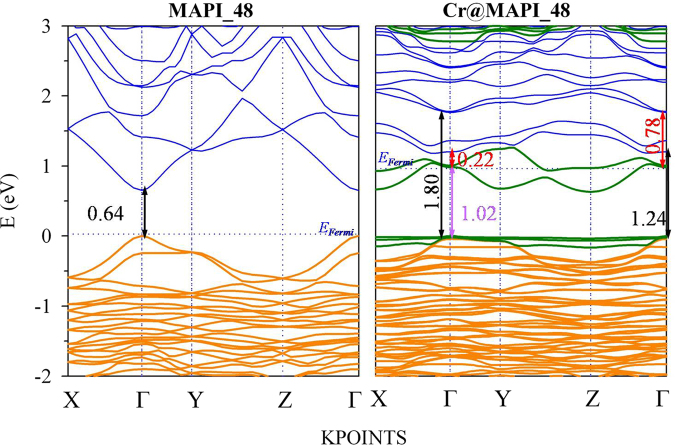
Projected band structure of MAPI_48 and Cr@MAPI_48 perovskites calculated with PBEsol+SOC along to main energy differences measured at Γ point (black, violet and red stand for VB-CB, VB-IGB and IGB-CB energy differences, respectively). Orange, Blue and Green colors stand for the main contribution of I, Pb and Cr atoms. The zero of energy has been set at the valence band energy, while blue dotted line is representing the Fermi level (*E*_*Fermi*_).

The projected density of states and band structures of the native perovskite (MAPI_48) calculated with PBEsol+SOC are shown in Figs 3 and 4, respectively; spin-up and spin-down bands and DOS are not given separately as electronic spin is no longer a good quantum number when spin-orbit coupling is included. Results for larger supercells (MAPI_96*a* and MAPI_96*b*) are not discussed here because important differences were not found respect to MAPI_48. Results here presented for unsubstituted MAPI are in agreement with our previous work^51^. At PBEsol+SOC theory level, MAPI perovskite yields a direct bandgap of 0.64 eV at Γ-point, which is highly underestimated compared with the experimental one (1.68 eV)^73^. A detailed inspection of the projected density of states (Fig. 3) shows that the VB is mainly due to I atoms (*5p*-orbitals) with some contributions of Pb (*6s*-orbitals), while the conduction band is mainly contributed by Pb (*6p*-orbitals), with a small contributions of I (5*p*-states). The bands from methylammonium cation are located at a lower energy region (below −4.0 eV). Besides, some information can be obtained from the shape of the bands and the effective masses can be computed. Note that around Γ-point (Fig. 4), the valence band is somewhat flatter than conduction one, which would point out that electrons are lighter than holes. The effective masses of holes (*m*^*h*^) and electrons (*m*^*e*^) have been calculated by parabolic fitting of the VB and the CB along the directions Γ-X, Γ-Y and Γ-Z. We found that *m*^*h*^/*m*_0_ = 0.19/0.15/0.18 (*m*_0_ is the free electron mass) and *m*^*e*^/*m*_0_ = 0.16/0.10/0.16 along Γ-X/Γ-Y/Γ-Z directions. Both *m*^*h*^ and *m*^*e*^ yield quite similar values, in agreement with the ambipolar transport behavior experimentally reported. Further, smaller *m*^*e*^ values also agree with larger diffusion constant for electrons than for holes, which would be useful for an efficient photovoltaic conversion^74,75^.

**Figure 5:**
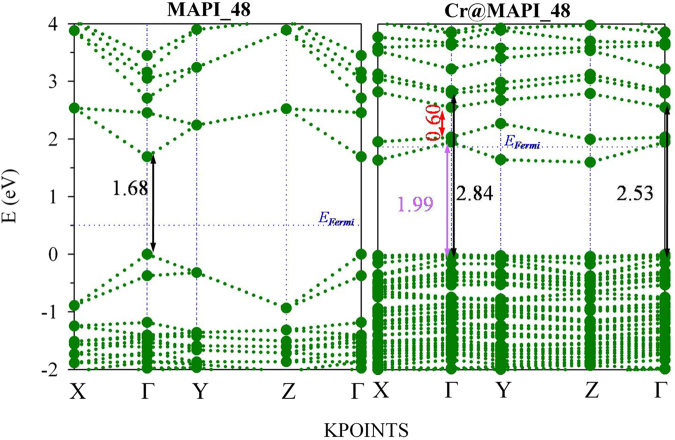
Band structure plots of MAPI_48 and Cr@MAPI_48 calculated within *G*_0_*W*_0_ + SOC approach along to main energy differences measured at Γ point (black, violet and red stand for VB-CB, VB-IGB and IGB-CB energy differences, respectively). The zero of energy has been set at the valence band energy, while blue dotted line is representing the Fermi level (*E*_*Fermi*_).

**Figure 6:**
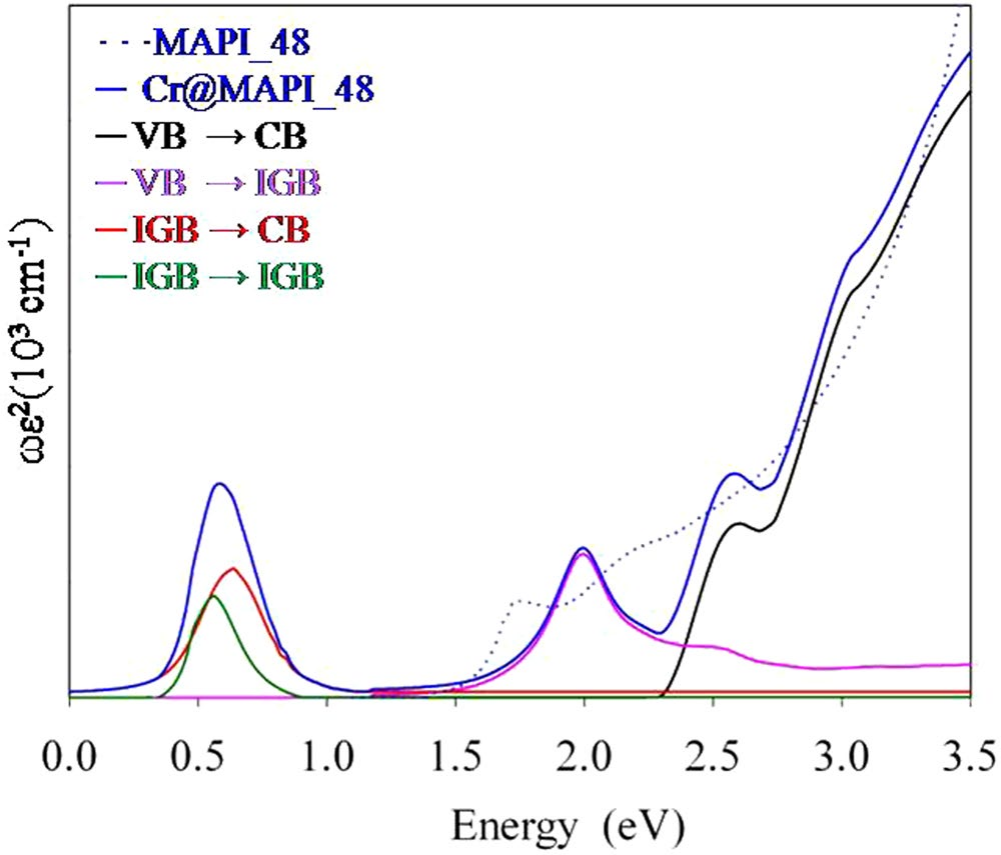
Imaginary part of the dielectric function *ε*^*(2)*^, multiplied by the frequency *ω*, for MAPI_48 and Cr@MAPI_48 compounds. Partial contributions due to VB-CB, VB-IGB, IGB-CB and IGB - IGB electronic transitions for Cr@MAPI_48 have been also included.

The bandgap calculated with PBEsol+SOC for MAPI_48 is considerably underestimated in comparison to experimental value. Hence, aiming at obtaining accurate bandgap values and, in general, electronic structure related properties, quasiparticle band structures were calculated via the *G*_0_*W*_0_+SOC approach (see Fig. 5). The calculated bandgap of 1.68 eV agrees well with the experimental reports^73^. Both PBEsol+SOC and *G*_0_*W*_0_+SOC methods give similar qualitative band structure pattern. Based on quasiparticle energies, a rigid shift equal to 1.04 eV over empty bands would be applied to correct bandgap energies calculated with PBEsol+SOC (see Fig. 6). Standard DFT methods could thus provide a qualitative description of the band structure; even though more accurate methods (such as *G*_0_*W*_0_+SOC) are needed for a correct description on the electronic structure.

Figures 3 and 4 also show the calculated projected density of states and band structure Cr@MAPI_48 perovskite with PBEsol+SOC approximation. Inspection of the structure shows that when one Pb atom is substituted by Cr, two new levels appear in the host semiconductor bandgap which mainly comprise Cr *3d*-orbitals. These new levels fulfill the requirements to play as IGB, *i.e*. this band is partially filled (the Fermi level crosses it), isolated from both CB and VB and has a small dispersion (the IGB has the higher bandwidth = 0.57 eV at Y-point). Moreover, three occupied bands also due to Cr *3d*-orbitals appear overlapping the VB, with energy somewhat higher than the last occupied bands due to I atoms. As discussed above, Cr atom is placed in an octahedral configuration coordinated to six I atoms, with an average I-Cr bond length equal to 2.85 Ǻ. The density of states analysis shows that in the valence band maximum, no significant overlap between Cr *3d* and I *5p-*orbitals exists, which indicates less covalent bonding between Cr and I in CrI_6_ octahedra (Fig. 3). According to crystal field theory, in a high spin configuration as is surely the case here the interaction between the transition metal (Cr) and I in a octahedral symmetry would cause a split of the Cr 3*d*-orbitals into a low-energy *t*_*2g*_ triplet (due to *d*_*xy*_, *d*_*xz*_ and *d*_*yz*_) and a high-energy *e*_*g*_ doublet (${d}_{{z}^{2}}$ and ${d}_{{x}^{2}-{y}^{2}}$)^76^. Thus, the three occupied bands of Cr atoms overlapping the VB would be related to the *t*_*2g*_ triplet, while the IGB could be due to the *e*_*g*_ doublet. However, a detailed inspection of the projected density of states shows that the IGB band comprises a mix of ${d}_{{z}^{2}}$, ${d}_{{x}^{2}-{y}^{2}}$ and ${d}_{yz}$), whilst the three occupied bands are mainly due to *d*_*xy*_ and *d*_*xz*_ orbitals with some contributions of *d*_*yz*_ and ${d}_{{z}^{2}}$ orbitals (Fig. S2). As seen in Fig. 2, I-Cr bonds can be grouped into three pairs with bond lengths ≈2.92 Ǻ, 2.86 Ǻ and 2.76 Ǻ, which implies that the octahedral coordination of Cr atom is distorted leading to a more complex distribution of *d-*orbitals. At Γ-point, main energy differences related with this IGB are 1.02 eV (labeled as VB-IGB) and 0.22 eV (IGB-CB transitions), which lead to a VB-CB energy difference equal to 1.24 V, which is considerably larger than that found for MAPI_48 (0.64 eV). Upon Pb replacement by Cr, the CB and VB do not change their nature significantly; the main contributions to them are still due to I *5p*-orbitals and Pb *6p*-orbitals, respectively. Though the shape of CB is more affected by Cr presence.

Although standard DFT methods could be adequate to provide a qualitative understanding of the electronic structure, we need to overcome their bandgap underestimation. For MAPI_48 perovskite this problem has been fixed through a rigid shift over to empty states to match the bandgap calculated by using *G*_0_*W*_0_+SOC approach. A similar procedure has been here applied for Cr@MAPI_48, where different the rigid shifts have been applied to correct the VB-IGB and IGB-CB energy differences (see Figs S5 and S6). Figure 5 plots the band structure for Cr@MAPI_48 by using *G*_0_*W*_0_+SOC approach along to main energy differences measured at Γ-point. Again, both PBEsol+SOC and *G*_0_*W*_0_+SOC approaches give similar qualitative band structure patterns. As expected, the band structure of Cr@MAPI_48 calculated with *G*_0_*W*_0_+SOC is also characterized by an IGB crossed by the Fermi level, well isolated from both VB (VB-IGB energy difference =1.99 eV at Γ point) and CB (IGB-CB energy difference = 0.60 eV), and with a bandwidth = 0.63 eV at Y-point. The lowest direct gap between the IGB and the CB is 0.41 eV at Y point, while the lowest indirect gap (0.28 eV) is measured between the Y and Γ points. Both gaps might decrease somewhat due to thermal vibrations; our previous evaluation has shown that band edge positions can be blurred ≈0.1 eV^62^. Thus, there would be no mixing between IGB and CB, *i.e*., the existence of a gap between the IGB and the CB. In addition, several works have demonstrated the formation of an IGB, whose energy differences with the conduction band are ≈0.25 eV^77–79^. In a similar way as in the PBEsol calculation, three flat bands are also noted in the valence band maximum, which should also be due to Cr atoms. Based on quasiparticle energies, a rigid shift of 0.97 eV and 0.38 eV should be applied to VB-IGB and IGB-CB, respectively, which leads to a rigid shift equal to 1.35 eV for VB-CB transitions. After that, main energy differences (VB-IGB, IGB-CB and VB-CB) calculated with PBEsol+SOC agree well with those ones obtained with *G*_0_*W*_0_+SOC (see Figs 6, S5 and S6).

Optical absorption features of MAPI and Cr-substituted MAPI perovskites have been assessed through the imaginary part of the dielectric function (see Fig. 6). Again, based on *G*_0_*W*_0_+SOC results, DFT energies have been corrected to fix the bandgap underestimation. For the native MAPI perovskite (MAPI_48), the absorption edge starts at ≈1.60 eV, which corresponds to the bandgap of the material (1.68 eV). For Cr@MAPI_48 there is an enhancement of the absorption features below the bandgap of the host semiconductor. As expected, for any IGB material (such as Cr@MAPI_48) the first absorption peak is due to electronic transitions between the different states forming the in gap band. Although these transitions contribute to the overall absorption process, they are not directly related with the generation of photocurrent of the device^80^. Absorption spectra of Cr@MAPI is characterized by three absorption peaks at ≈0.60 eV (this peak overlaps the IGB-IGB transition), 2.00 eV and 2.60 eV, which are principally due to IGB-CB transitions, VB-IGB transitions and VB-CB transitions, respectively. All peaks due to electronic transitions involving the IGB are at energies previously forbidden for the pure semiconductor.

**Figure 7:**
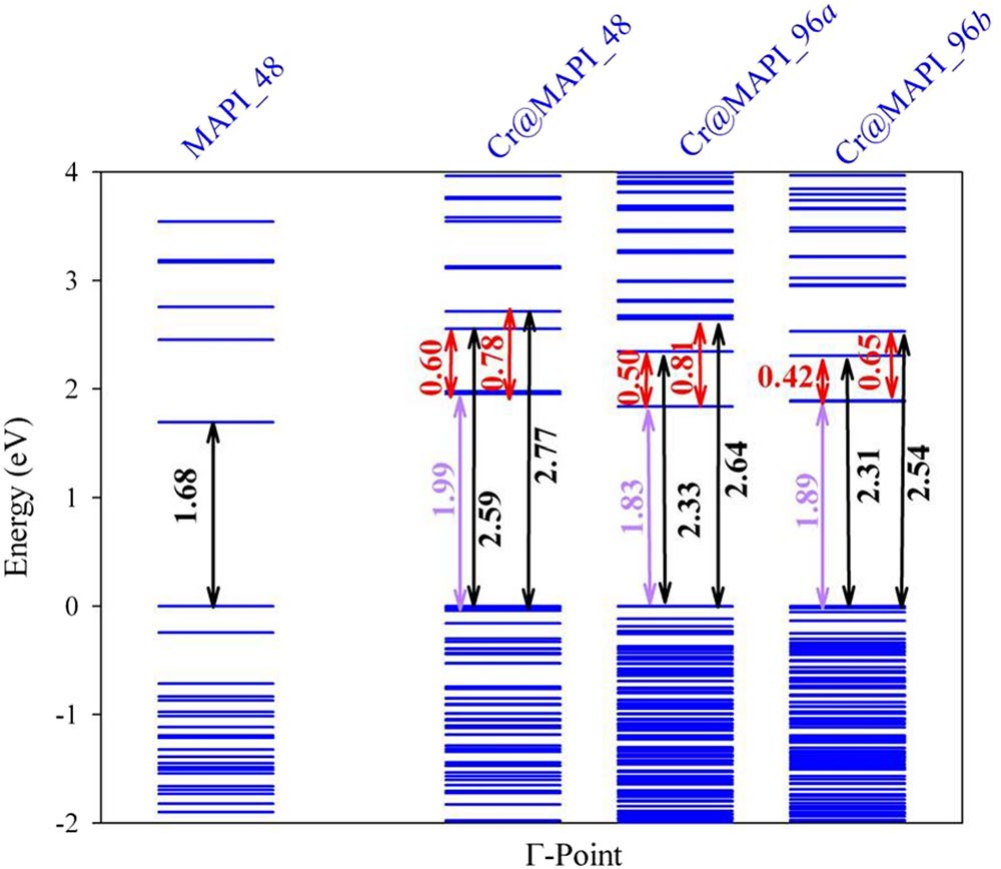
Eigenvalues calculated for MAPI and Cr@MAPI perovskites at Γ-point obtained after applying a rigid shift (based on *G*_0_*W*_0_+SOC approach) over PBEsol + SOC. Main energy differences measured at Γ-point (black, violet and red stand for VB-CB, VB-IGB and IGB-CB energy differences, respectively) are also shown. (Cr@MAPI_96*a*) and MAPI_96*b* (Cr@MAPI_96*b*) stand for the unit cell with 48 atom, 96 atoms supercell with *a ≈ b ≈ c* and 96 atoms 2 × 1 × 1 supercell, respectively. The zero of energy has been set at the valence band energy.

Finally, we have also assessed the influence of Cr concentration on the electronic structure. Thus, the electronic band structure and density of states of Cr@MAPI_96*a* and Cr@MAPI_96*b* were also obtained by using PBEsol+SOC approach. As discussed for Cr@MAPI_48, the Cr-substitution leads to the presence of a new band in the gap of the host semiconductor (in gap band) as well as three new occupied bands (see Figs S3, S4, S7 and S8 for more details). Aimed at obtained a direct connection between our calculations and the improvement of the absorption of perovskites here studied, in Fig. 7 we report the main energy differences measured at Γ-point obtained after applying the scissor operator above discussed for the native MAPI perovskite (MAPI_48) as well as for Cr-substituted MAPI perovskites at two different Cr quantities (x = 0.25 for Cr@MAPI_48 and x = 0.125 for Cr@MAPI_96*a* and Cr@MAPI_96*b*). As seen in Fig. 6, for the pristine perovskite, the absorption edge would start at 1.60 eV–1.70 eV, which corresponds to the bandgap of the material (1.68 eV). For the Cr@MAPI_48 compound, the absorption would be extended up to 0.60 eV. Thus, two peaks at 0.60 eV and 0.78 eV can be expected due to IGB-IGB and IGB-CB transitions, while the absorption peak due to VB-IGB will be located at around 1.99 eV. Finally, VB-CB transitions will start to appear at 2.59 eV. Upon lower Cr quantity, the absorption would be extended up to 0.50 eV/0.42 eV according to electronic structure of Cr@MAPI_96*a*/Cr@MAPI_96*b*. The decrease in the amount of Cr also allows that VB-IGB transitions emerge at lower energies: 1.83 eV/1.89 eV for Cr@MAPI_96*a*/Cr@MAPI_96*b*. The absorption edge due to VB-CB transitions would be slightly red-shifted up to ≈2.32 eV. Results here exposed illustrate that an improved sunlight absorption extended below 1.0 eV can be expected due to new electronic transitions across the IGB. This would enable the use of Cr-MAPI perovskites in more efficient photovoltaic devices.

## Conclusions

In this work, we have studied the effect of Pb replacement by Cr in methylammonium lead halide perovskite (MAPI): CH_3_NH_3_Pb_1−x_Cr_x_I_3_ (Cr@MAPI) by using standard DFT and many-body (in *G*_0_*W*_0_ approach) calculations along spin-orbit coupling. As with Pb in the pristine MAPI perovskite, Cr atoms are placed in an octahedral environment of I atoms. This CrI_6_ octahedron shows a bond length alternation, wherein I-Cr bonds can be grouped into three pairs with bond lengths ≈2.92 Ǻ, 2.86 Ǻ and 2.76 Ǻ. The energetics of the substitution reaction is compatible with the possibility of obtaining experimentally an intermediate band material through substitution of Pb by Cr. Based on DFT calculations, MAPI perovskite yields a direct bandgap of 0.64 eV at Γ-point, which is considerably underestimated, as usual, in comparison to the experimental value. The band structure calculated with *G*_0_*W*_0_+SOC approach yields a bandgap value (1.68 eV) for MAPI, which agrees well with experimental data. Hence, in this work a rigid shift based on *G*_0_*W*_0_+SOC calculations has been applied over empty bands to correct bandgap energies obtained with standard DFT methods. As concerns the electronic structure of Cr@MAPI perovskite, DFT calculations point out that Cr *3d-*orbitals induce the formation of a new partially filled band in the bandgap of the host semiconductor, which fulfills the requirements needed to be defined as an IGB. In addition, Cr *3d*-orbitals also lead to the presence of three new occupied bands with energy somewhat larger than the highest occupied valence band contributed mainly by of I atoms. The full relaxation of Cr@MAPI perovskites provided structures with slightly distorted CrI_6_ octahedral coordination that leads to a more complex distribution of *d-*orbitals. Thus, the IGB is due to a mix of ${d}_{{x}^{2}-{y}^{2}}$ ${d}_{{z}^{2}}$, and ${d}_{yz}$ orbitals. Similarly, for Cr@MAPI_48, the same kind of calculations based on *G*_0_*W*_0_+SOC approach has been also applied to provide and accurate electronic structure description. Our results indicate that the absorption edge of the native perovskite would start at 1.60 eV–1.70 eV, corresponding to its bandgap. For Cr@MAPI perovskites the absorption would be extended below ≈0.60 eV (the value will depend on the Cr concentration) due to the effect of Cr-substitution on the electronic structure. Moreover, new absorption peaks would be noted due to VB-IGB at ≈1.80 eV. Thus, these results highlight that an improved sunlight absorption could be expected due to new electronic transitions across the in gap band.

### Theoretical Details

The calculations here exposed have been performed using the Vienna *ab initio* simulation package (VASP)^81,82^. Firstly, structural optimizations were performed using the PBEsol functional^83^. PBEsol is a revision of the PBE functional, specifically customized for solids, that has been shown to yield structural data in agreement with experiment^7^. The projector augmented wave (PAW) pseudopotential^84,85^ was used with an energy cutoff of 400 eV. The Brillouin zone has been sampled using a 4 × 4 × 4 Γ-centered grid. Forces and total energies are converged to 0.01 eV Å^−1^and 10^–4^ eV, respectively. The crystal structure of CH_3_NH_3_PbI (MAPI) perovskite was modeled by means of a unit cell with 48 atoms belonging to the *Pnma* (62) symmetry space group (from now on MAPI_48), which is the appropriate one for low temperature studies^14^. Since the experimental crystal structure determination using XRD does not yield the position of the H atoms, the optimized structure at optB88-vdW previously published by our group was selected as starting structure^51^. In addition, two larger 96-atoms supercells were built from the previous one: (*a*) through symmetry operations aimed at obtaining one unit cell with lattice parameters *a* *≈* *b* *≈* *c* (MAPI_96*a*); and (*b*) by doubling lattice parameter *a* (MAPI_96*b*). The crystal structures of Cr-substituted perovskites CH_3_NH_3_Pb_1−x_Cr_x_I_3_ (Cr@MAPI) were then built by replacing in each of these cells one Pb by Cr, which led to Cr concentrations of x = 0.25 for the unit cell with 48 atoms (Cr@MAPI_48) and x = 0.125 for larger supercells (Cr@MAPI_96*a* and Cr@MAPI_96*b*). The crystal structure labeled as MAPI_96*a* will thus lead to similar Cr-Cr distances along the three lattice parameters. Based on optimized geometries, an energy cutoff of 700 eV and a *k*-point mesh of 8 × 8 × 8 (6 × 6 × 6) Γ-centered grid for 48-atoms unit cell (for 96 atoms supercells) have been used for electronic structure calculations. Due to the presence of heavy atoms (Pb), electronic structure calculations with spin-orbit coupling (SOC), *i.e*., PBEsol+SOC, were always carried out.

In order to overcome the underestimation of the bandgap of standard-DFT methods, we employ many-body perturbation theory in *GW* approximation^59^. As said, *GW*+SOC has been recently applied to MAPI perovskites yielding accurate bandgap in agreement with experimental data^13,55,60^. The *GW* approach is based on a dynamic dielectric screening of the Coulomb potential, while the electronic self energy is approximated by a convolution in terms of the Green’s function *G* and the screened interaction *W*. In this paper, *GW* calculations were carried out to correct DFT eigenvalues without further interactions, *i.e., G*_0_*W*_0_ approach^59^, wherein the calculation starts from DFT eigenvalues and eigenfunctions to obtain many-body *GW* self energy. This method yields successfully agreement with experiment results for intermediate band materials, within the limits of the approach^86^. Since *GW* approximation is very time consuming, quasi particle energies here reported were only computed for MAPI_48 and Cr@MAPI_48 compounds. *G*_0_*W*_0_ approach was carried out also including SOC, with an energy cutoff of 300 eV, a Γ-centered 2 × 2 × 2 k-point to sample the Brillouin zone and 3000 bands. Quasiparticle calculations with denser k-mesh (4 × 2 × 2, 4 × 2 × 4 and 3 × 3 × 3) were also carried out for MAPI_48 yielding bandgap differences roughly 0.1 eV.

Optical properties have been assessed through the absorption coefficient derived from the dielectric functions as implemented in VASP code. The imaginary part has been calculated as the sum of independent transitions between Kohn-Sham states, without local field effects, while the real part was obtained from the imaginary part by the Kramers-Krönig relations. Because of the imaginary part of the dielectric function is obtained as a sum over independent transitions, it can be separated into the contributions from different transitions. This analysis was carried out by using a home-modified version of the original OPTICS code developed by J. Furthmueller^87^.

## Electronic supplementary material

Supplementary Information
