# Author Correction: Influence of chromium hyperdoping on the electronic structure of CH_3_NH_3_PbI_3_ perovskite: a first-principles insight

**DOI:** 10.1038/s41598-018-25173-6

**Published:** 2018-05-03

**Authors:** Gregorio García, Pablo Palacios, Eduardo Menéndez-Proupin, Ana L. Montero-Alejo, José C. Conesa, Perla Wahnón

**Affiliations:** 1 Instituto de Energía Solar, ETSI Telecomunicación, Universidad Politécnica de Madrid, 28040 Madrid, Spain; 2 Departamento de Tecnología Fotónica y Bioingeniería, ETSI Telecomunicación, Universidad Politécnica de Madrid, Ciudad Universitaria, s/n, 28040 Madrid, Spain; 3 Departamento de Física aplicada a las Ingenierías Aeronáutica y Naval, ETSI Aeronáutica y del Espacio, Universidad Politécnica de Madrid, Pz. Cardenal Cisneros, 28040, 3 Madrid, Spain; 4 Grupo de Modelización de Materiales, Departamento de Física, Facultad de Ciencias, Universidad de Chile, Las Palmeras, 3425, 780-0003 Ñuñoa, Santiago Chile; 5 Instituto de Catálisis y Petroleoquímica, Consejo Superior de Investigaciones Científicas, Marie Curie 2, 28049 Madrid, Spain

Correction to: *Scientific Reports* 10.1038/s41598-018-20851-x, published online 06 February 2018

In the original version of this Article, Perla Wahnon was incorrectly affiliated with ‘Departamento de Física aplicada a las Ingenierías Aeronáutica y Naval, ETSI Aeronáutica y del Espacio, Universidad Politécnica de Madrid, Pz. Cardenal Cisneros, 3, 28040 Madrid, Spain.’. The correct affiliations are listed below.

^1^Instituto de Energía Solar, ETSI Telecomunicación, Universidad Politécnica de Madrid, 28040, Madrid, Spain.

^2^Departamento de Tecnología Fotónica y Bioingeniería, ETSI Telecomunicación, Universidad Politécnica de Madrid, Ciudad Universitaria, s/n, 28040 Madrid, Spain.

This error has now been corrected in the PDF and HTML versions of the Article and in the accompanying Supplementary Information file.

