# Supplementary material for: Influence of chromium hyperdoping on the electronic structure of CH3NH3PbI3 perovskite: a first-principles insight
Source: Sci Rep. 2018 Feb 6;8:2511. doi: 10.1038/s41598-018-20851-x (PMC5802816; doi:10.1038/s41598-018-20851-x)
Supplement: Supplementary file 1 — Supplementary Information [file 41598_2018_20851_MOESM1_ESM.docx]

**Influence of chromium hyperdoping on the electronic structure of CH_3_NH_3_PbI_3_ perovskite: a first-principles insight**

Gregorio García,^1,2*^ Pablo Palacios,^1,3^ Eduardo Menéndez-Proupin,^4^ Ana L. Montero-Alejo,^4^ José C. Conesa^5^ and Perla Wahnón^1,2^

^1^Instituto de Energía Solar, ETSI Telecomunicación, Universidad Politécnica de Madrid, 28040, Madrid, Spain

^2^Departamento de Tecnología Fotónica y Bioingeniería, ETSI Telecomunicación, Universidad Politécnica de Madrid, Ciudad Universitaria, s/n, 28040 Madrid, Spain

^3^Departamento de Física aplicada a las Ingenierías Aeronáutica y Naval, ETSI Aeronáutica y del Espacio, Universidad Politécnica de Madrid, Pz. Cardenal Cisneros, 3, 28040 Madrid, Spain.

^4^Group of NanoMaterials, Departamento de Física, Facultad de Ciencias, Universidad de Chile, Las Palmeras 3425, 780-0003 Ñuñoa, Santiago, Chile

^5^Instituto de Catálisis y Petroleoquímica CSIC, Marie Curie 2, 28049 Madrid, Spain

* Corresponding author: [ggmoreno@etsit.upm.es](mailto:ggmoreno@etsit.upm.es) (G.G.)

**Supporting Information**


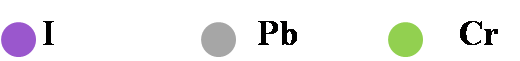


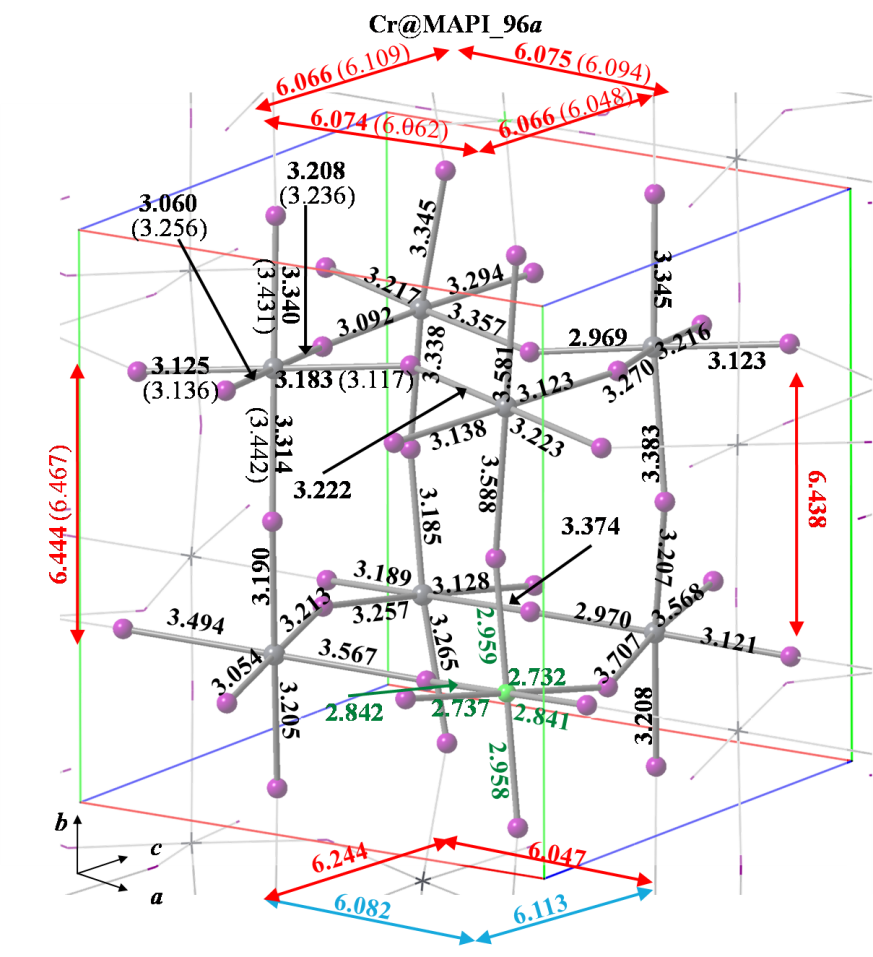


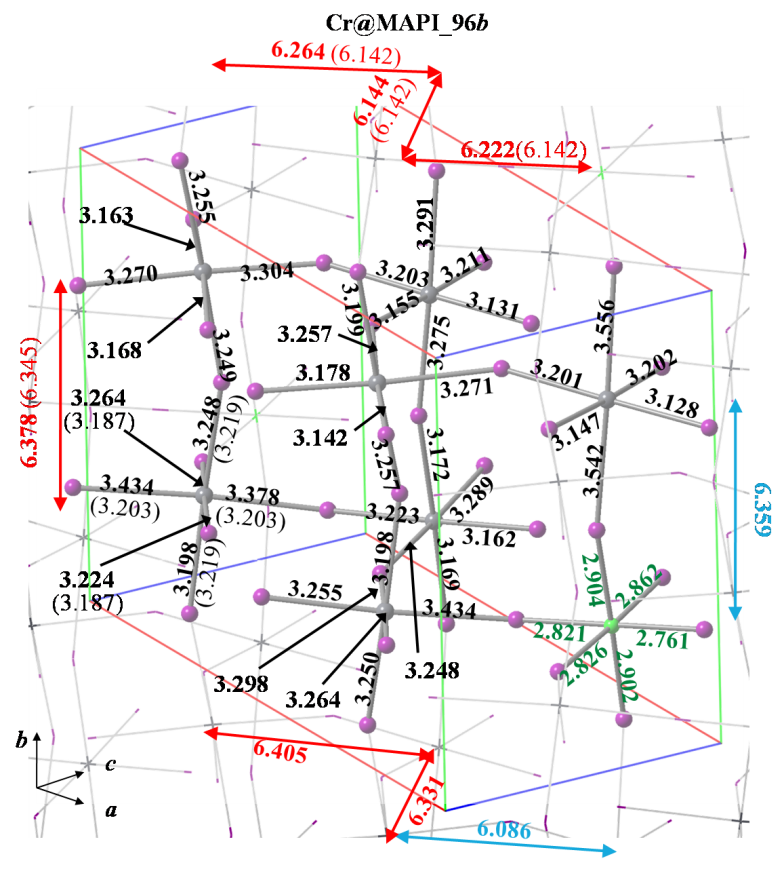


**Figure 1S.** Optimized structures (with PBEsol+SOC approach)of Cr@MAPI_96*a*(96 atoms supercell with *a≈b≈c*) and Cr@MAPI_96*b* (96 atoms with 2x1x1 supercell), along with bond lengths of I-Pb (black), I-Cr (green), Pb-Pb (red) and Pb-Cr (blue). Values in parenthesis stand for those bond lengths for the optimized structures of the native MAPI (which has all Pb atoms equivalent). For clarity, Pb-Pb and Pb-Cr bond distances are displayed outside of the unit cell. Methylammonioum cations have been omitted for simplicity.


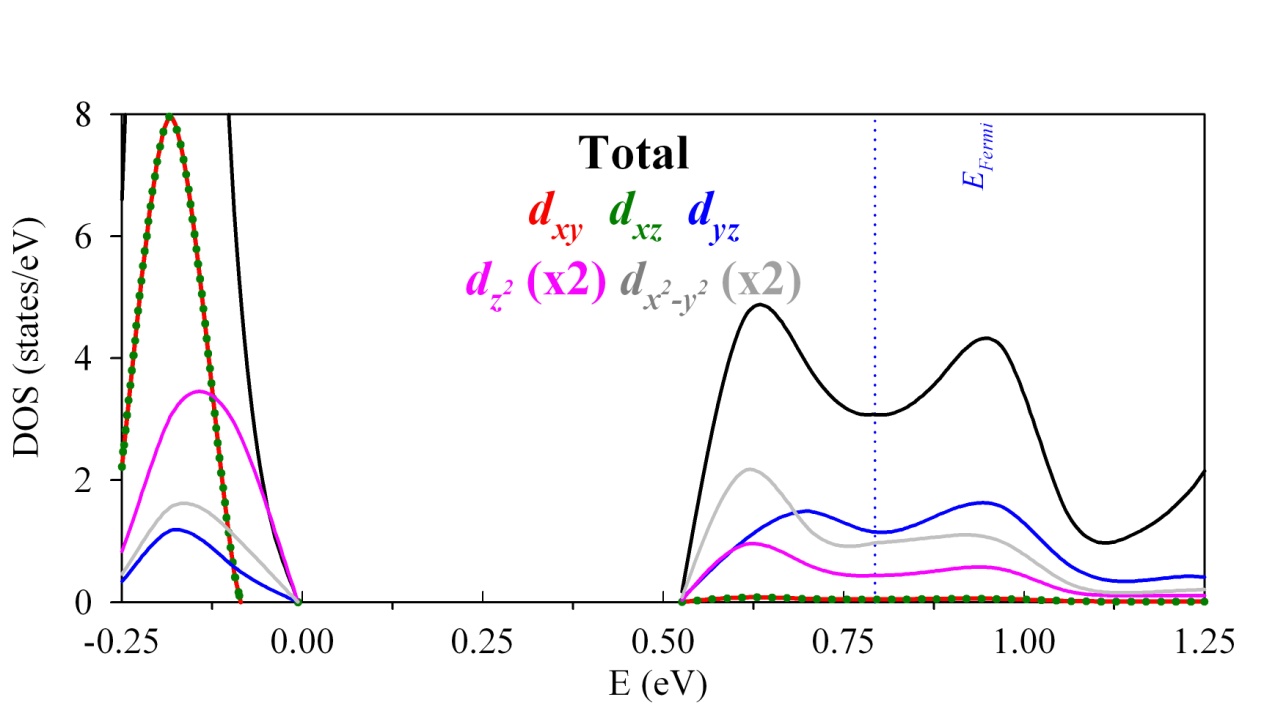


**Figure 2S.** Projected density of states of Cr@MAPI_48 perovskite calculated with PBEsol+SOC, showing the contributions of different Cr 3*d-*orbitals. The zero of energy has been set at the valence band top, while the blue dotted line is representing the Fermi level (*E_Fermi_*).

**
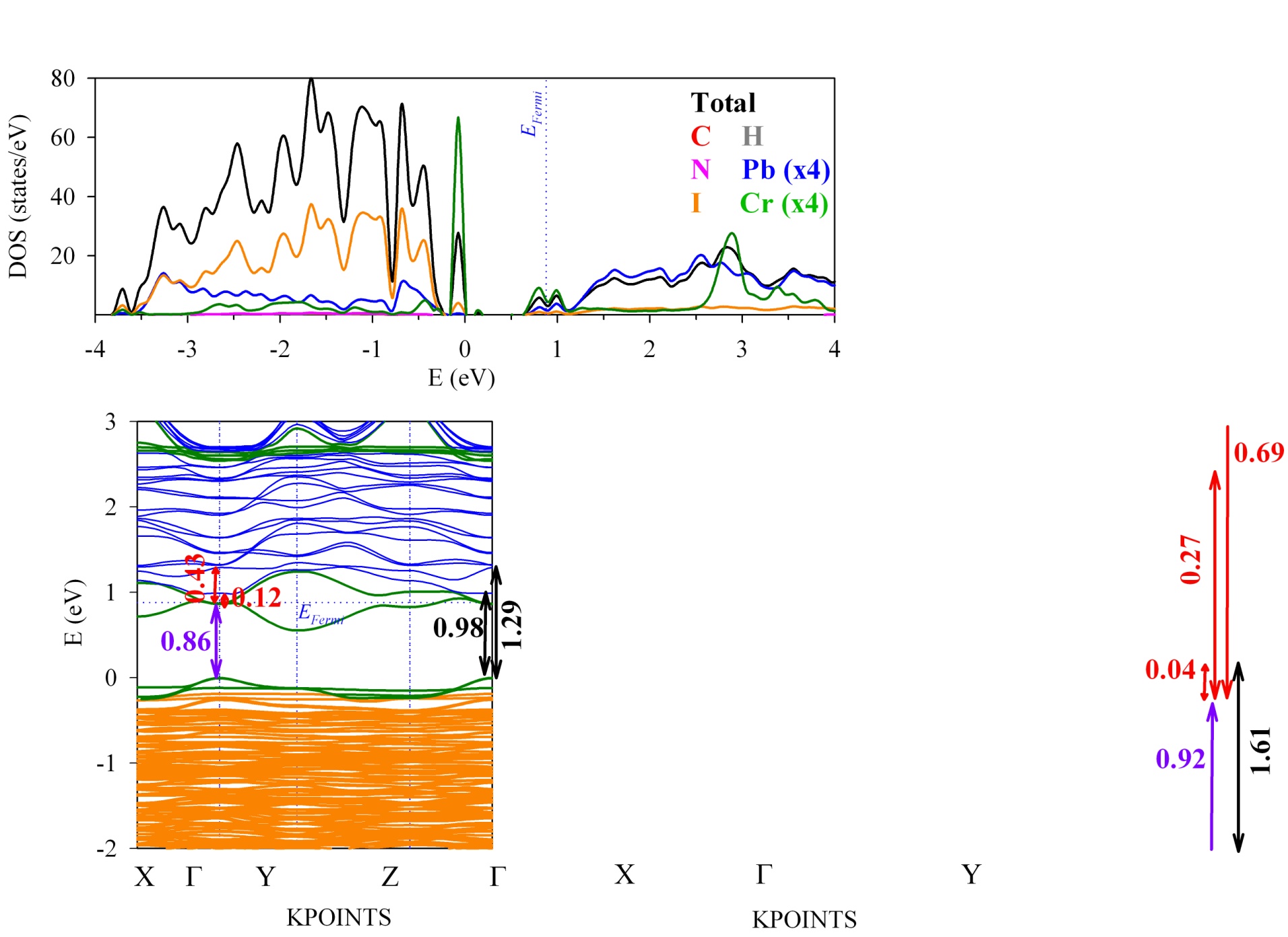
**

**
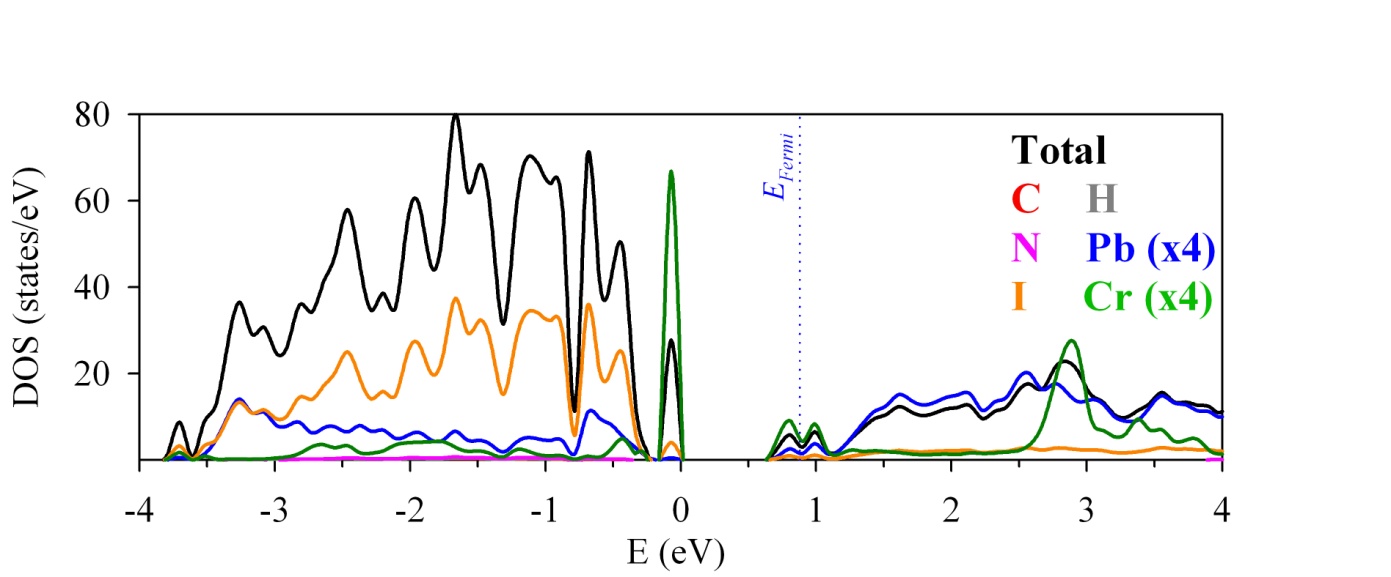
**

**Figure 3S.** Up: Projected band structure (up),including the main energy differences measured at Γ point (orange, blue and green colors stand for the main contributions of I, Pb and Cr atoms, respectively; black, violet and red colors stand for VB-CB, VB-IGB and IGB-CB energy differences, respectively); Down: Projected density of states (down) of Cr@MAPI_96*a* perovskite calculated with PBEsol+SOC. The zero of energy has been set at the valence band top, while the blue dotted line is representing the Fermi level (*E_Fermi_*).

**
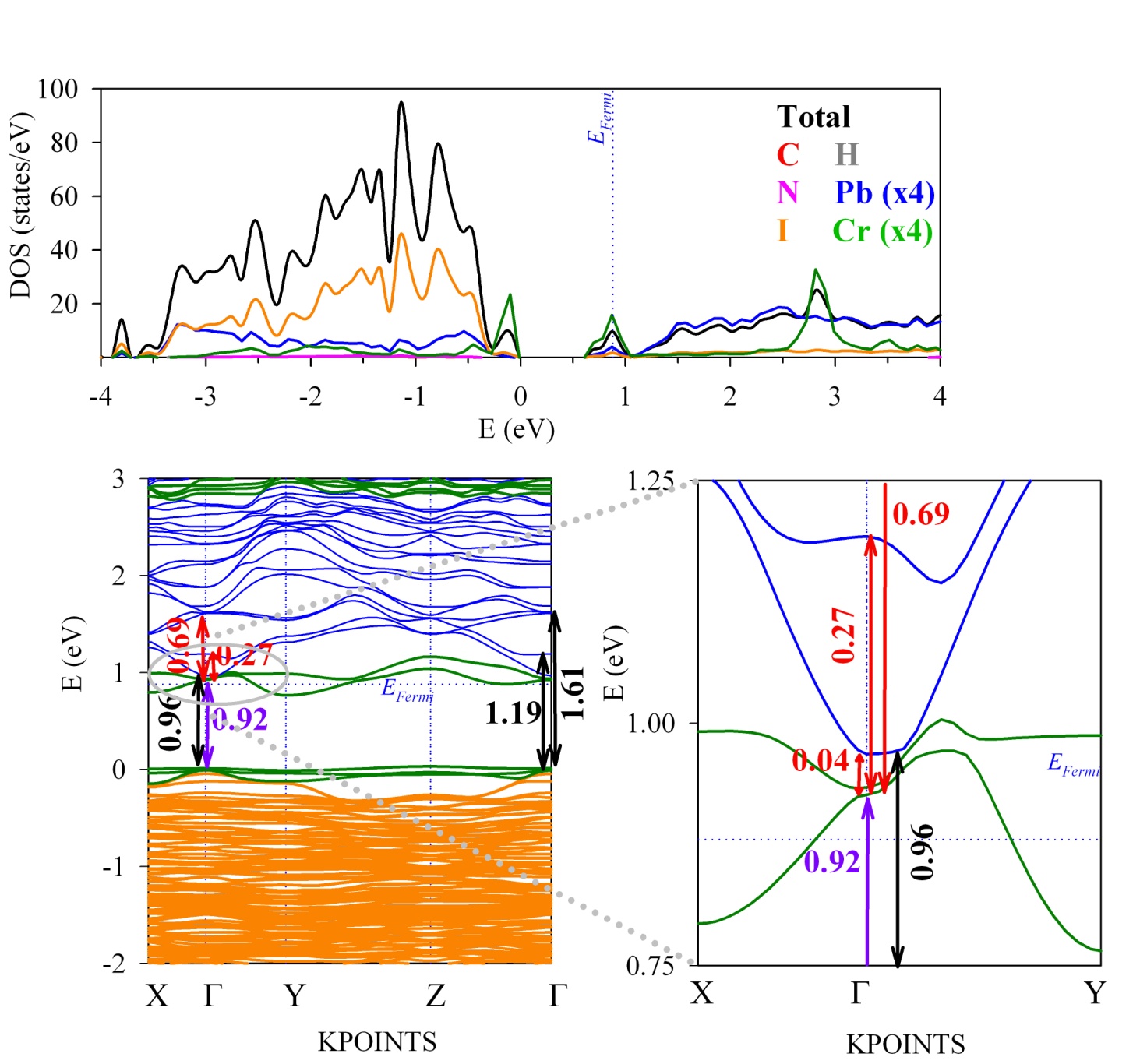
**

**
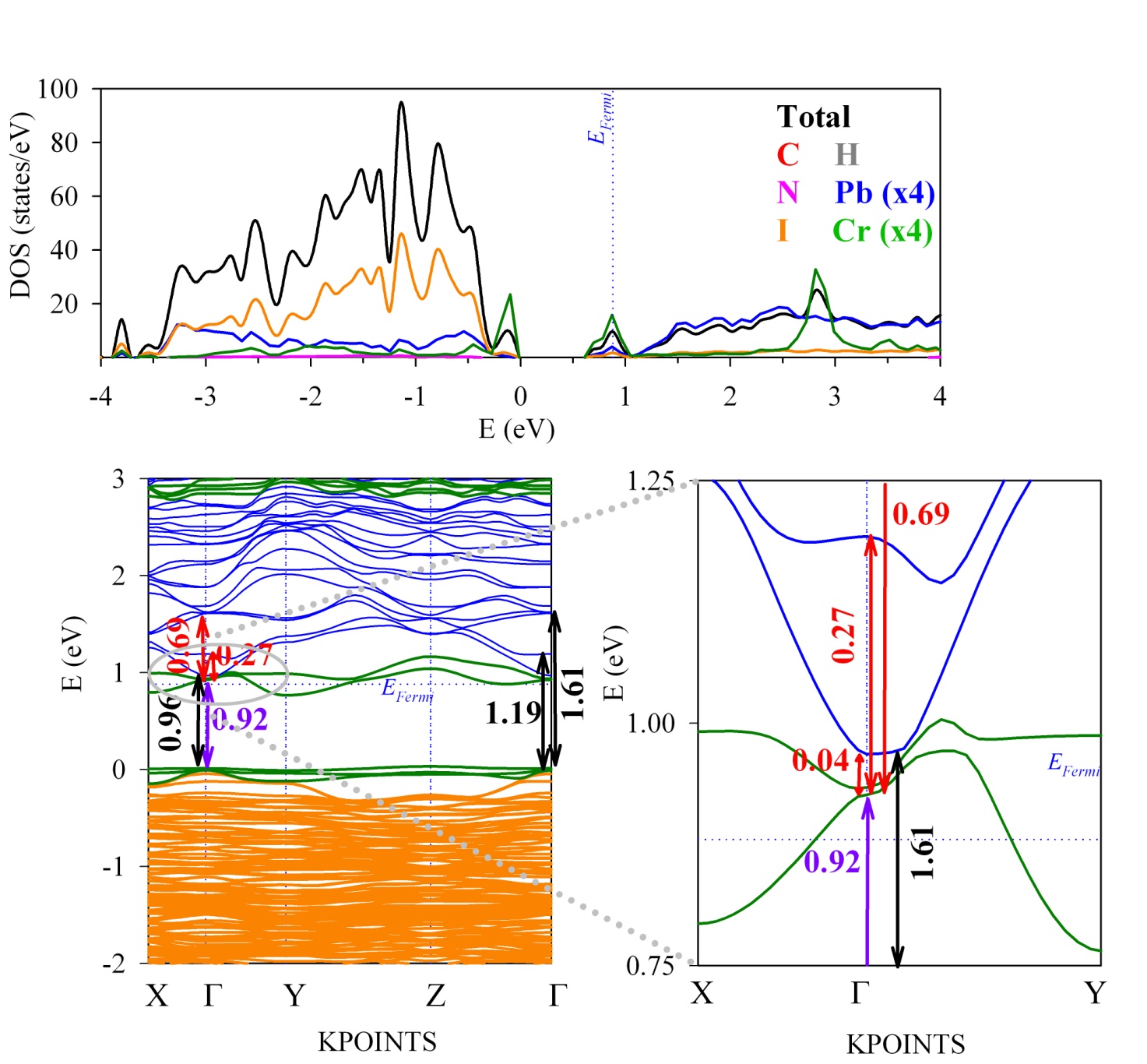
**

**Figure 4S.** Up: Projected band structure (up) including the main energy differences measured at Γ point (orange, blue and green colors stand for the main contribution of I, Pb and Cr atoms, respectively; black, violet and red colors stand for VB-CB, VB-IGB and IGB-CB energy differences, respectively); Down: Projected density of states (down) of Cr@MAPI_96*b* perovskite calculated with PBEsol+SOC. The zero of energy has been set at the valence band top, while the blue dotted line is representing the Fermi level (*E_Fermi_*).

**
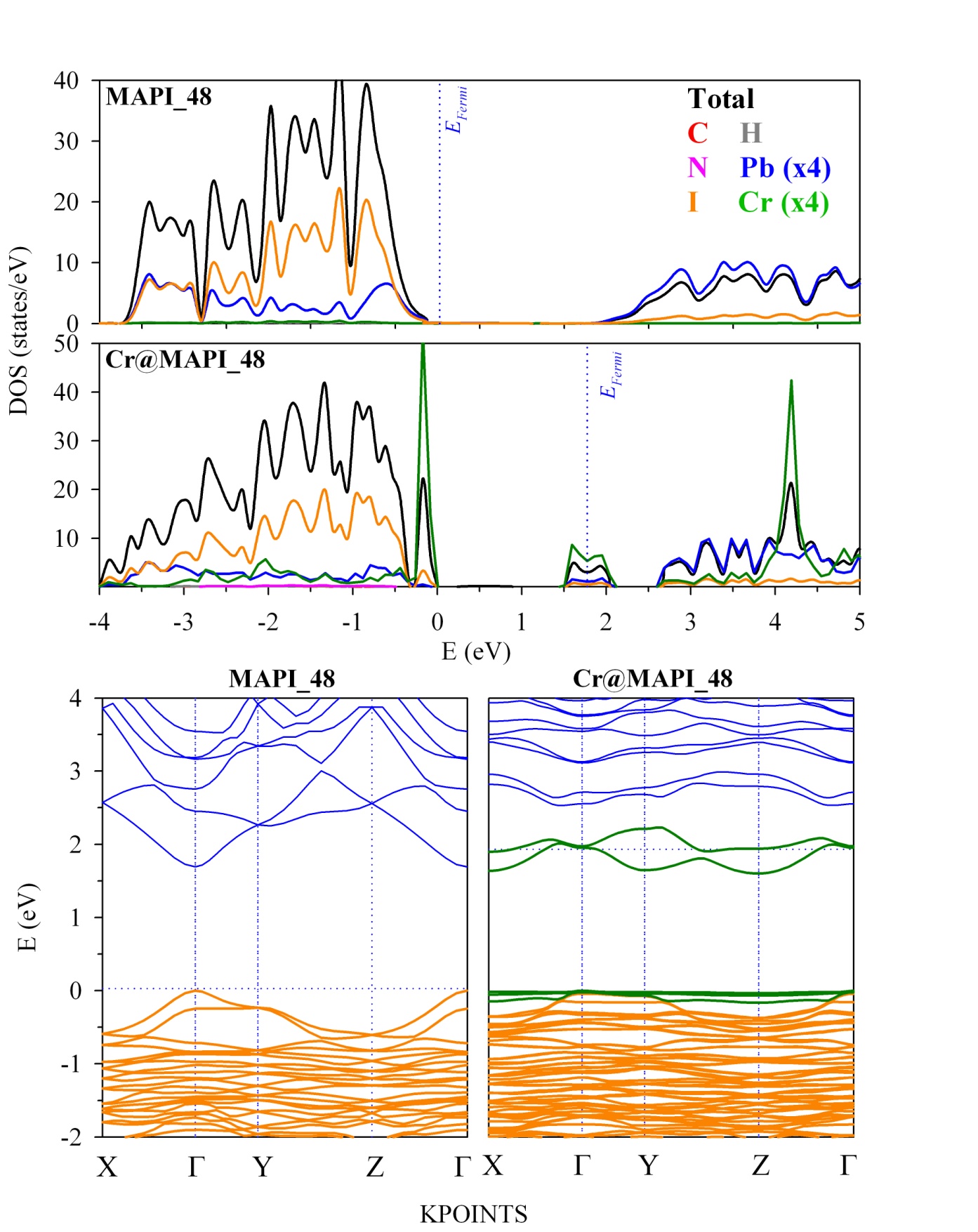
**

**Figure 5S.** Projected Density of States of MAPI_48 and Cr@MAPI_48 obtained after applying a rigid shift (based on *G_0_W_0_*+SOC approach) over PBEsol+SOC. The zero of energy has been set at the valence band energy, while blue dotted line is representing the Fermi level (*E_Fermi_*).

**
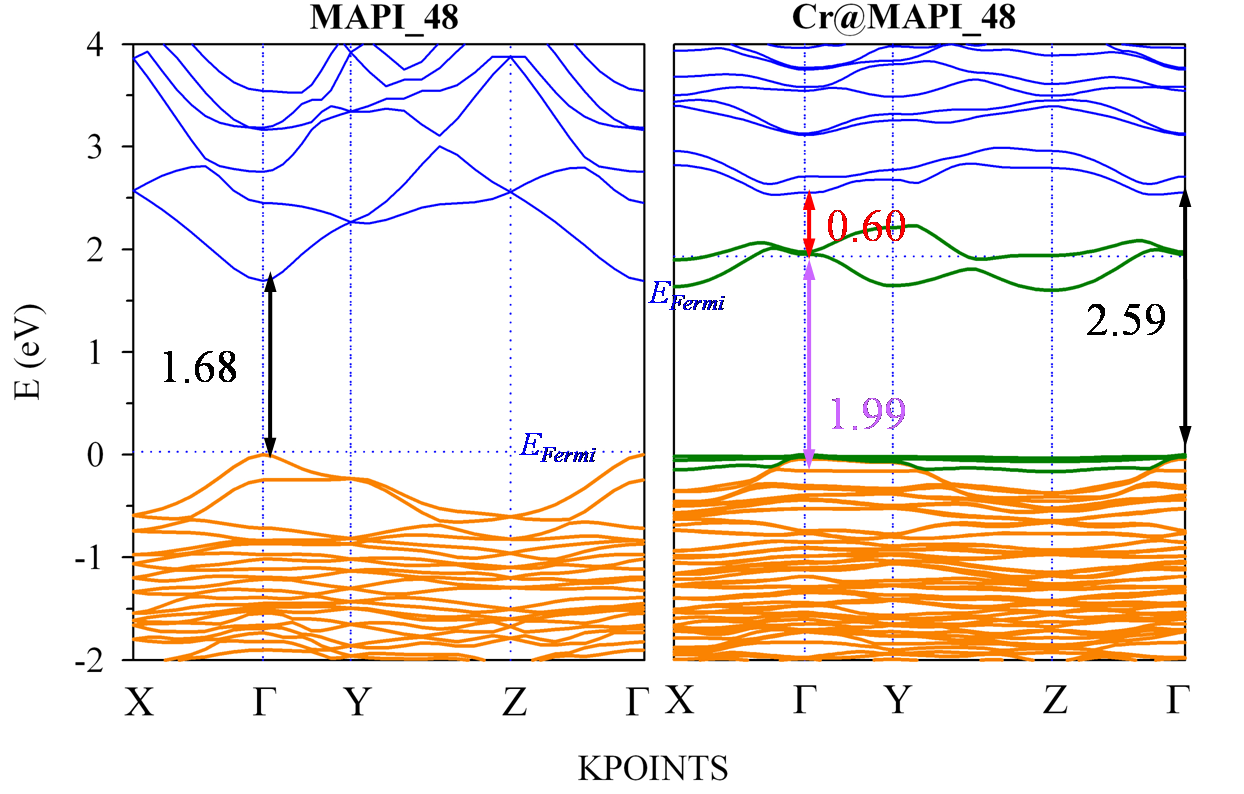
**

**Figure 6S.** Projected band structure of MAPI_48 and Cr@MAPI_48 perovskites obtained after applying a rigid shift (based on *G_0_W_0_*+SOC approach) over PBEsol+SOC along to main energy differences measured at Γ point (black, violet and red stand for VB-CB, VB-IGB and IGB-CB energy differences, respectively). Orange, Blue and Green colors stand for the main contribution of I, Pb and Cr atoms. The zero of energy has been set at the valence band energy, while blue dotted line is representing the Fermi level (*E_Fermi_*).

**
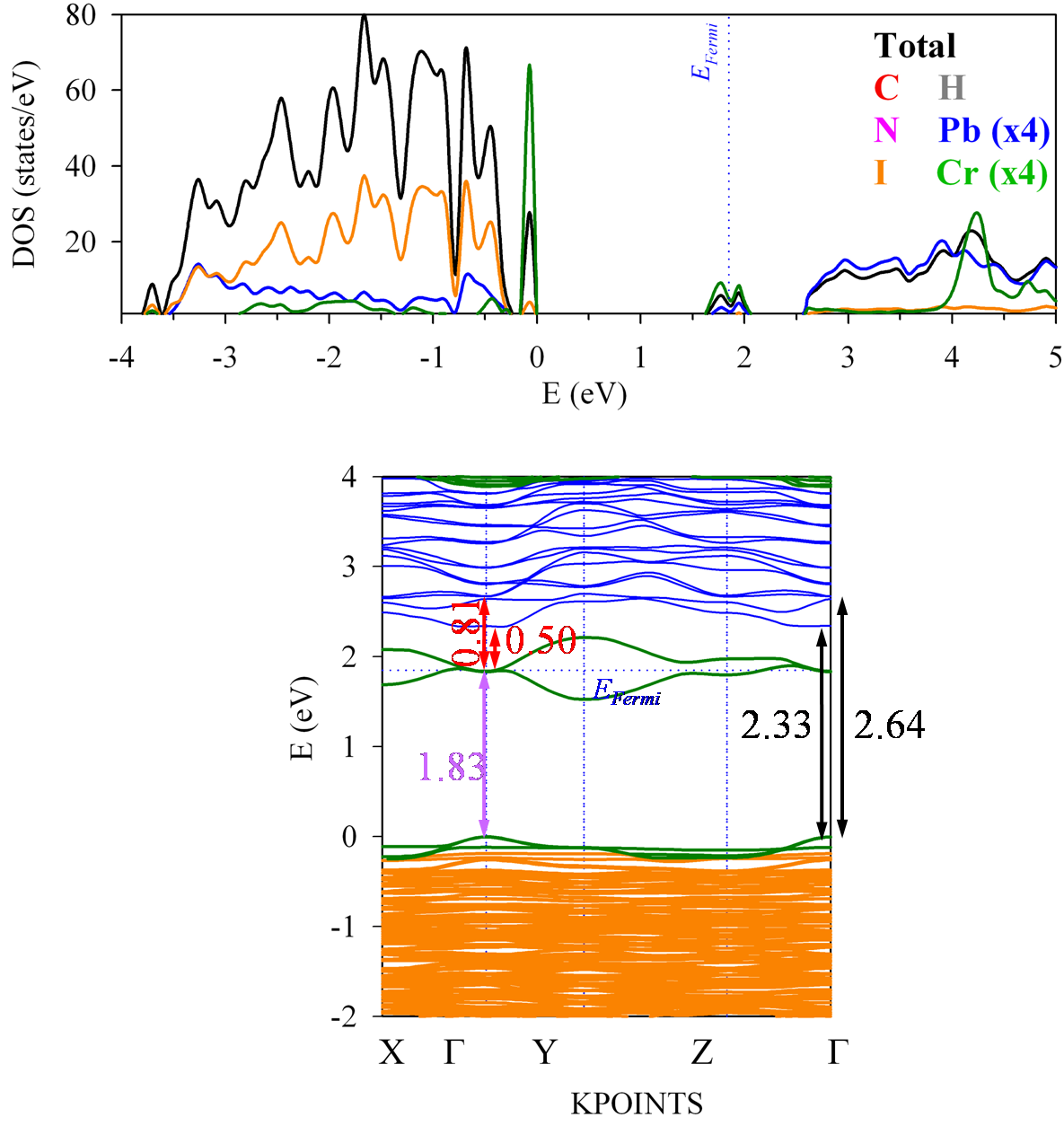
**

**
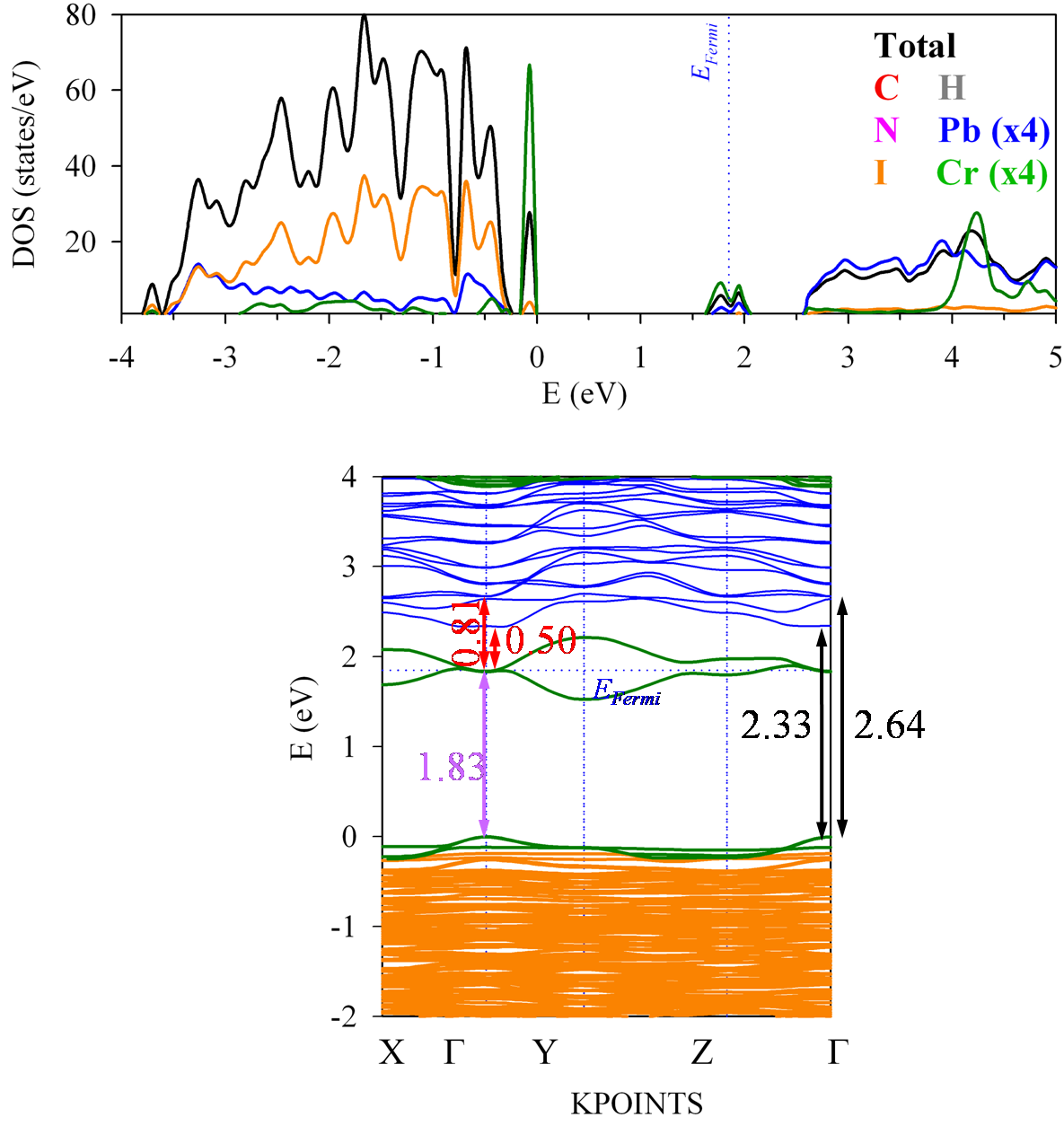
**

**Figure 7S.** Up: Projected band structure (up),including the main energy differences measured at Γ point (orange, blue and green colors stand for the main contributions of I, Pb and Cr atoms, respectively; black, violet and red colors stand for VB-CB, VB-IGB and IGB-CB energy differences, respectively); Down: Projected density of states (down) of Cr@MAPI_96*a* perovskite obtained after applying a rigid shift (based on *G_0_W_0_*+SOC approach) over PBEsol+SOC. The zero of energy has been set at the valence band top, while the blue dotted line is representing the Fermi level (*E_Fermi_*).

**
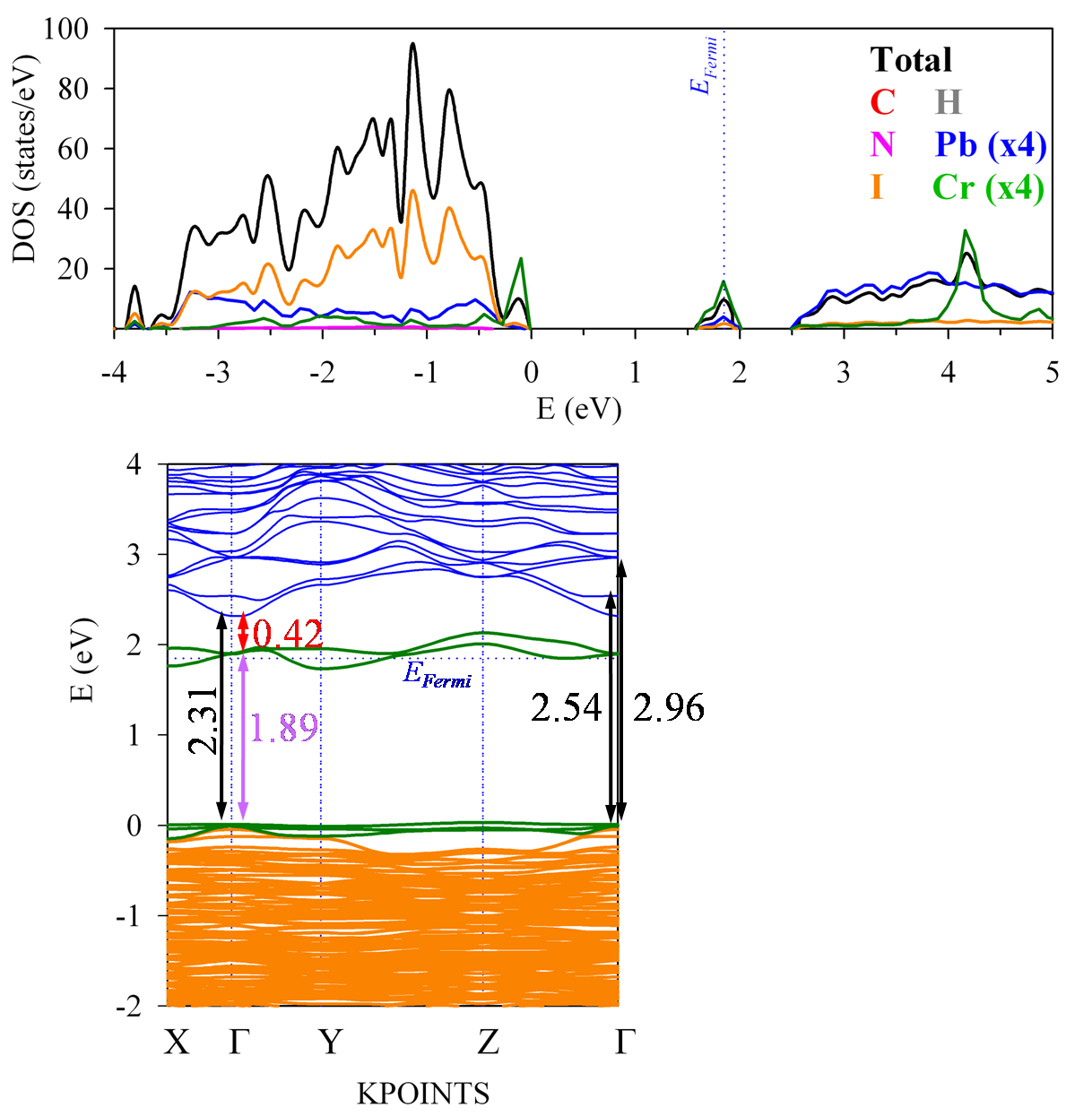
**

**
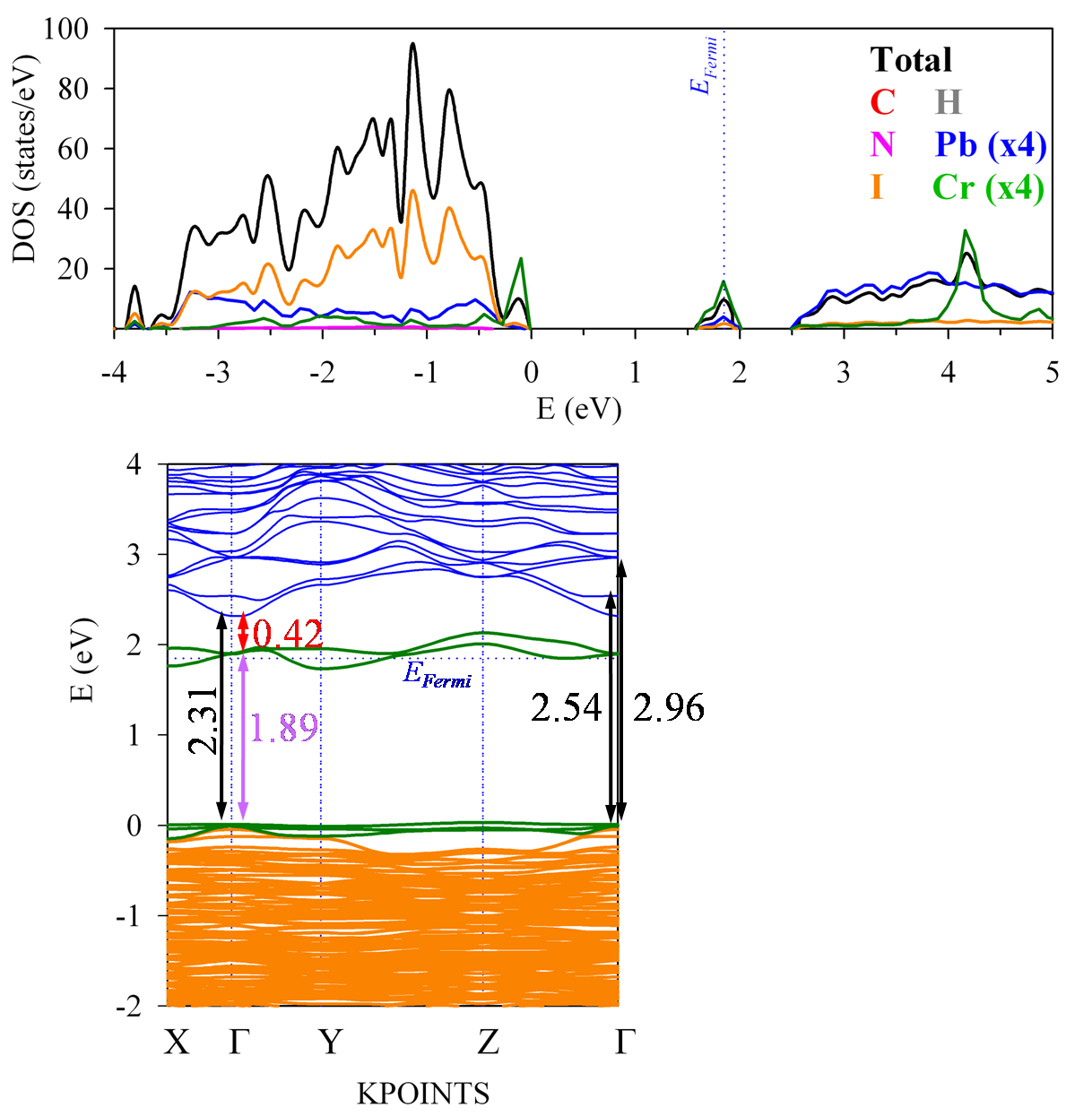
**

**Figure 8S.** Up: Projected band structure (up) including the main energy differences measured at Γ point (orange, blue and green colors stand for the main contribution of I, Pb and Cr atoms, respectively; black, violet and red colors stand for VB-CB, VB-IGB and IGB-CB energy differences, respectively); Down: Projected density of states (down) of Cr@MAPI_96*b* perovskite obtained after applying a rigid shift (based on *G_0_W_0_*+SOC approach) over PBEsol+SOC. The zero of energy has been set at the valence band top, while the blue dotted line is representing the Fermi level (*E_Fermi_*).
